# Optimization of water-saving irrigation decision using dynamic spatiotemporal graph neural networks

**DOI:** 10.1038/s41598-026-54923-0

**Published:** 2026-05-28

**Authors:** Yilong Fang, Junlin Zheng, Xiaojun Shen, Chao Zhang, Kadambot H.M. Siddique

**Affiliations:** 1 College of Water Conservancy, Shenyang Agricultural University, Shenyang, 110866 China; 2 College of Water Conservancy Engineering, Tianjin Agricultural University, Tianjin, 300392 China; 3 Liaoning Hongcheng Architectural Planning and Design Institute Co., Ltd, Shenyang, 124100 China; 4 The UWA Institute of Agriculture, The University of Western Australia, Perth, WA 6009 Australia

**Keywords:** Spatiotemporal graph neural network, Dynamic water demand prediction, Irrigation decision optimization, Multimodal data fusion, Climate sciences, Engineering, Environmental sciences, Hydrology, Water resources

## Abstract

Water-saving irrigation is critical for improving water use efficiency and ensuring sustainable food production. However, accurate prediction of farmland water demand remains difficult because soil, crop, and meteorological processes interact across space, depth, and time. To address this problem, we develop an AI-STGNN-based irrigation decision framework in which AI-STGNN serves as the core predictive model. The main methodological contribution is a dynamic three-dimensional (3D) graph that represents vertical soil heterogeneity and time-varying hydrological connectivity, while channel-wise meteorological attention and multi-scale temporal memory are incorporated as agro-hydrology-specific enhancements. On the held-out 2021–2022 test set, AI-STGNN achieved RMSE, MAE, and R² values of 3.63 vol%, 2.88 vol%, and 0.89, respectively, reducing RMSE by 7.2% relative to STGCN and by 16.7% relative to LSTM. Under heavy rainfall, its RMSE increased by 45%, compared with 59–70% for the baseline models. In the two-season field comparison across three representative farms, the AI-STGNN-guided strategy reduced irrigation water use by 12.3–15.7%, increased crop yield by 3.5–4.2%, and improved water use efficiency by 13.7–17.8% relative to conventional empirical irrigation. Because the field comparison covers only two growing seasons and three sites, these practical gains should be interpreted as preliminary rather than definitive. Overall, the proposed framework provides a physically informed, data-driven basis for dynamic water-demand prediction and irrigation scheduling in the studied Midwest cropping system.

## Introduction

 Agricultural irrigation is the largest consumer of global freshwater resources, yet traditional irrigation practices often exhibit low water use efficiency. In the context of long-term climate change and growing populations, addressing water scarcity through improved on-farm water management has become a critical challenge for sustainable agriculture. Consequently, optimizing water-saving irrigation by accurately adapting to short-term weather variability and seasonal patterns is essential for improving water productivity and ensuring food security, making data-driven precision irrigation a central component of modern agricultural management^1^.

A core requirement of precision agriculture is the accurate prediction of crop water demand and the optimization of irrigation schedules. These tasks rely on a detailed understanding of farmland water cycles, which are driven by meteorological conditions and shaped by soil heterogeneity and crop water physiology, and exhibit multi-scale nonlinear coupling^2^. Traditional process-based hydrological models can represent these mechanisms to some extent, but they require extensive parameterization, incur high computational costs, and are often impractical for large-scale or real-time applications^3^.

Data-driven methods have emerged as promising alternatives. Early approaches used Random Forests (RF), Support Vector Machines (SVM), and Recurrent Neural Networks (RNN) to predict soil moisture and crop water demand, achieving modest improvements in time-series modeling^4^. However, these models generally rely on local or single-point observations, limiting their ability to capture spatial interactions among farmland units. Long Short-Term Memory (LSTM) models can capture long-term dependencies but struggle to account for heterogeneous meteorological influences on evapotranspiration^5^. Attention mechanisms have been introduced to identify key meteorological drivers, yet they remain limited in representing nonlinear interactions and multi-scale effects, and their performance often declines under extreme weather conditions^6^.

Recent studies have further extended this direction from static prediction toward adaptive irrigation decision support. Optimization-based irrigation management under uncertainty has been explored to improve water-saving decision-making at the farm level^7^. Climate-responsive irrigation adaptation has also received increasing attention under changing environmental conditions^8^. In parallel, recent studies have emphasized uncertainty-aware irrigation water allocation and dynamic regulation^9^, as well as intelligent irrigation scheduling strategies that combine advanced sequence modeling with adaptive control mechanisms^10^. These developments indicate that future irrigation systems require not only improved prediction accuracy, but also stronger adaptability, uncertainty awareness, and decision-oriented integration.

Graph Neural Networks (GNNs) have emerged as a powerful framework for modeling spatially distributed agricultural and hydrological systems by jointly capturing node dependencies and temporal dynamics. For example, Taccari et al.^11^ used graph structures to model spatiotemporal dependencies among farmland units, improving predictions of soil moisture and surface and groundwater levels. Roudbari et al.^12^ combined graph convolution with temporal models to capture hydrological correlations at both field and basin scales, demonstrating strong performance in water forecasting and drought and flood monitoring. Similarly, Wang et al.^13^ applied a Spatiotemporal Graph Convolutional Network (STGCN) to soil moisture forecasting, achieving superior long-term accuracy compared with LSTM and RF, while Raza et al.^4^ showed that integrating graph convolution with temporal modeling improved flow prediction in the Colorado River Basin. Recent work on heterogeneous graph structures has further demonstrated the value of exploiting multi-type node and edge relationships for representation learning^14^. Although that study focused on hyperspectral band selection rather than hydrological prediction, its emphasis on structurally informed graph representation provides useful methodological motivation for the dynamic edge formulation adopted in this study.

Despite these advances, most existing GNN-based hydrological models rely on predefined, static adjacency matrices, with edge weights fixed during training. Such static graph structures are unable to represent time-varying hydraulic connections driven by rainfall, evapotranspiration, and crop water use, thereby limiting model robustness and generalizability^15^. Furthermore, many studies adopt two-dimensional (2D) graph representations that focus solely on horizontal spatial relationships while neglecting the vertical heterogeneity of soil layers^16^. Recent studies have shown that vertical structures play a critical role in soil water storage and transport processes, and ignoring this dimension can lead to incomplete representations of farmland hydrological dynamics^17^. To address spatial complexity, Bentivoglio et al.^18^ proposed multi-scale graph structures incorporating grid-based adjacency and cross-scale interactions; however, these approaches still lack the ability to dynamically adapt graph topology in response to changing environmental conditions.

To address the above challenges, this study develops the Agricultural Irrigation Spatiotemporal Graph Neural Network (AI-STGNN) within a unified irrigation decision framework. The study makes one primary methodological contribution and two supporting system-level contributions. First, AI-STGNN introduces a dynamic three-dimensional (3D) graph that explicitly represents vertical soil heterogeneity, interlayer water transfer, and time-varying hydrological connectivity. Unlike conventional static 2D graph models, the graph structure is updated through attention-guided edge weights driven by soil moisture gradients and meteorological forcing. Intuitively, an edge becomes stronger when two adjacent field units or soil layers are more hydrologically coupled, either because moisture redistribution between them is active or because they are responding to the same rainfall forcing. It becomes weaker when their local water states evolve more independently, allowing the graph to preserve stable background adjacency while adapting local interaction strength over time. Second, the framework incorporates two adapted components that are tailored to the agro-hydrological setting rather than claimed as standalone novelties: a channel-wise meteorological attention module for separating the effects of heterogeneous weather drivers, and a multi-scale temporal memory module for capturing both gradual moisture evolution and abrupt disturbances. Third, these components are embedded in a closed-loop irrigation decision framework that links spatiotemporal prediction to constrained irrigation scheduling. Table 1 summarizes the differences between AI-STGNN and representative STGNN-based models.

**Table 1:** Comparison of AI-STGNN with representative STGNN-based models.

Method	Graph Type	Dimension	Meteorological Handling	Temporal Modeling
STGCN	Static	2D	Aggregated	Single-scale
LSTM-GNN	Static	2D	Aggregated	Sequential
AI-STGNN	Dynamic	3D	Channel-wise attention	Multi-scale memory

## Materials and methods

For clarity, all major mathematical symbols are defined when they first appear in the corresponding methodological formulations.

### Study area

The datasets used in this study were obtained from the International Soil Moisture Network (ISMN)^19^ and farmland meteorological observations released by the United States Department of Agriculture (USDA)^20^. The study area covers major agricultural regions in the U.S. Midwest, including 15 representative irrigated farms in Nebraska, Iowa, and Kansas (40°–42° N, 96°–99° W). These farms primarily cultivate corn and soybean. Together, they provide limited regional diversity in soil properties, local topographic settings, and management conditions within the studied maize–soybean production system. Therefore, they offer a representative sample for the agro-climatic context considered in this study, but do not fully encompass the broader variability of other climatic regions, soil types, or irrigation systems. The datasets provide continuous observations from 2015 to 2022, with a daily temporal resolution and a spatial resolution corresponding to farmland units of approximately 50 m × 50 m. Each farmland unit corresponds to a single agricultural plot of approximately 50 m × 50 m and is represented as one graph node in the proposed framework. The node features are constructed by integrating the soil observations from ISMN sensors, the meteorological variables from USDA records, and the corresponding crop and environmental attributes associated with that plot. In this way, the spatial unit used in the dataset is directly aligned with the node definition in the graph model.

### Dataset composition

The dataset comprises 15 farmland units with continuous 8-year observations, covering 365 days per year (366 in leap years) and yielding 43,800 unit-day records. Remote sensing vegetation indices available at 16-day intervals were resampled to a daily scale using cubic spline interpolation to align them temporally with the other inputs^21^. Cubic spline interpolation was selected because it preserves smooth seasonal vegetation trajectories better than simple linear interpolation and is therefore more suitable for approximating gradual crop-growth dynamics between consecutive MODIS observations. Because cloud contamination produced a 12.3% missing rate in the original MODIS series, the vegetation-index time series were first smoothed and reconstructed using the Savitzky–Golay filter before temporal interpolation. These interpolated daily values were treated as auxiliary predictors rather than direct observations, and their uncertainty is acknowledged in the discussion of data limitations. A detailed description of all variables is provided in Table 2. The inclusion of multisource crop and environmental predictors is supported by recent studies showing that integrating spectral, thermal, and ancillary variables can improve soil-moisture and crop water-status estimation^22,23^.

**Table 2:** Detailed composition of the experimental dataset.

Data Type	Variables	Source	Temporal Resolution	Spatial Resolution	Sample Size	Missing Rate
Meteorological	Daily precipitation (mm)	USDA	Daily	Station	43,800	2.3%
	Daily mean temperature (°C)	USDA	Daily	Station	43,800	1.8%
	Daily max/min temperature (°C)	USDA	Daily	Station	43,800	1.8%
	Relative humidity (%)	USDA	Daily	Station	43,800	3.1%
	Wind speed (m/s)	USDA	Daily	Station	43,800	2.7%
	Solar radiation (MJ/m²/day)	USDA	Daily	Station	43,800	4.2%
Soil	Surface soil moisture (0–10 cm, %)	ISMN sensors	Daily	50 m×50 m	43,800	3.5%
	Root-zone soil moisture (10–40 cm, %)	ISMN sensors	Daily	50 m×50 m	43,800	4.1%
	Deep soil moisture (40–100 cm, %)	ISMN sensors	Daily	50 m×50 m	43,800	5.8%
	Soil temperature (°C)	ISMN sensors	Daily	50 m×50 m	43,800	3.2%
	Soil electrical conductivity (dS/m)	ISMN sensors	Daily	50 m×50 m	43,800	6.5%
	Soil texture (%)	USDA	Static	50 m×50 m	15	0%
	Soil organic matter content (%)	USDA	Static	50 m×50 m	15	0%
Crop	NDVI	MODIS MOD13Q1	16 days	250 m	2,738	12.3%
	EVI	MODIS MOD13Q1	16 days	250 m	2,738	12.3%
	LAI	Ground observation	Weekly	Farm	624	8.7%
	Crop growth stage	Farm records	Event-based	Farm	120	0%
	K_c_	FAO-56 standard	Growth stage	Crop type	–	0%
Topographic	DEM (m)	USGS	Static	30 m	15	0%
	Slope (°)	Derived from DEM	Static	30 m	15	0%

### Data preprocessing

#### Missing value handling

Missing meteorological and soil values were filled using a hybrid strategy: gaps ≤ 3 days were filled using linear interpolation^24^; gaps > 3 and ≤ 7 days were filled using a 7-day moving average; gaps > 7 days were removed. Remote sensing vegetation indices, which had a higher missing rate (12.3%) due to cloud contamination, were smoothed and reconstructed using the Savitzky–Golay filter. Although daily interpolation improves temporal consistency across multimodal inputs, it may introduce additional uncertainty because the original MODIS observations are available only at 16-day intervals. Therefore, the interpolated vegetation indices should be interpreted as smooth approximations of crop development rather than direct daily observations. A formal sensitivity analysis comparing cubic spline interpolation with alternative temporal interpolation schemes was not conducted in the present study, so the quantitative effect of that preprocessing choice cannot yet be stated. Because the MODIS-derived variables were used only as auxiliary predictors within a broader multimodal input set, we expect the model to be less sensitive to this choice than to the in-situ soil-moisture and meteorological inputs, but that expectation still requires dedicated verification. In this study, these variables were used as auxiliary predictors rather than sole decision variables, which reduces the risk that interpolation error alone dominates model predictions. Nevertheless, the uncertainty introduced by temporal interpolation remains a limitation of the present dataset and should be further examined in future studies using higher-frequency remote sensing or denser field observations.

#### Outlier detection

Outliers were identified using the 3σ rule, where values outside the range of mean ± 3 standard deviations were flagged. Additional physical constraints were imposed on bounded variables; specifically, soil moisture and relative humidity were restricted to the range of 0–100%. Detected outliers were corrected following the same strategy used for missing values.

A quantitative summary of outlier detection results is provided in Table 3. Across the 11 dynamic variables, a total of 7,227,000 records were screened. The 3σ criterion flagged 52,416 records, corresponding to 0.73% of all dynamic observations, while an additional 1,284 records (0.018%) were identified through physical boundary checks. The combined correction rate was therefore 0.75%, which is consistent with the expected data quality range of field-scale environmental sensor networks.

**Table 3:** Variable-level outlier detection statistics.

Variable	Total Records	3σ Flagged	Physical Boundary Flagged	Combined Rate (%)
Daily precipitation	657,000	2,103	0	0.32
Daily mean temperature	657,000	2,036	0	0.31
Relative humidity	657,000	3,942	312	0.64
Wind speed	657,000	3,285	0	0.50
Solar radiation	657,000	4,467	0	0.68
Surface soil moisture (0–10 cm)	657,000	4,599	658	0.80
Root-zone soil moisture (10–40 cm)	657,000	5,256	314	0.84
Deep soil moisture (40–100 cm)	657,000	7,749	0	1.18
Soil temperature	657,000	4,272	0	0.65
Soil electrical conductivity	657,000	9,341	0	1.42
NDVI (interpolated daily)	657,000	5,366	0	0.82
Total	7,227,000	52,416	1,284	0.75

Among all variables, the highest outlier rate was observed in soil electrical conductivity, at 1.42%, followed by deep soil moisture (40–100 cm), at 1.18%. Meteorological variables showed lower outlier rates overall, ranging from 0.31% for daily mean temperature to 0.68% for solar radiation. These results indicate that the abnormal observations were limited in proportion and did not dominate the data distribution.

To quantify the impact of outlier correction on model performance, a controlled validation experiment was conducted by comparing models trained on fully preprocessed data and on raw data with flagged values retained. The corrected-data model achieved a validation RMSE of 3.71 vol%, compared with 4.03 vol% for the raw-data variant, corresponding to a 7.9% reduction in prediction error. This result confirms that outlier preprocessing materially improves data quality and downstream predictive performance.

#### Data standardization

To eliminate scale differences among variables, all features were normalized using Z-score standardization^25^:

1 $$x_{{i,t,d}}^{{\mathrm{'}}} = \frac{{x_{{i,t,d}} - \mu _{d} }}{{\sigma _{d} }}$$

where $\:{x}_{i,t,d}$ is the original value of feature *\:d* at node *\:i* and time *\:t*, $\:{x}_{i,t,d}^{{\prime\:}}$ is the standardized value, and $\:{\mu\:}_{d}$ and $\:{\sigma\:}_{d}$ are the mean and standard deviation of feature *\:d*, respectively. To prevent data leakage, $\:{\mu\:}_{d}$ and $\:{\sigma\:}_{d}\:$ were computed only from the training set and then applied unchanged to the validation and test sets.

#### Spatial alignment

All datasets were spatially aligned to a uniform 50 m × 50 m farmland grid using geographic information systems (GIS). Meteorological station data were interpolated using inverse distance weighting (IDW), and remote sensing data with an original spatial resolution of 250 m were resampled to the target 50 m grid using bilinear interpolation. However, these spatial preprocessing steps may introduce additional uncertainty. In particular, resampling MODIS-scale observations from 250 m to 50 m may lead to mixed-pixel effects and local smoothing, especially in areas with heterogeneous surface conditions. Similarly, IDW interpolation may not fully capture fine-scale meteorological variability when station spacing is relatively sparse. Therefore, the spatially aligned variables should be interpreted as approximations of local conditions rather than exact measurements at the farmland-unit scale.

In this study, these resampled variables were used as part of a multimodal input system rather than as standalone decision variables, which reduces the likelihood that spatial interpolation error alone dominates the model outputs. Nevertheless, uncertainty associated with spatial resampling and interpolation remains a limitation of the present dataset and should be further examined in future studies using higher-resolution remote sensing products and denser in situ monitoring networks.

#### Time window construction

Time-series samples were generated using a sliding-window strategy^26^. Each sample used a 14-day historical window (T_in_ = 14) to predict soil moisture and crop water demand over the subsequent 7 days (T_out_ = 7). After preprocessing, the final model input is represented as a spatiotemporal feature tensor:

2 $$\:\begin{array}{c}X\in\: {\mathbb{R}} ^{N\times\:{T}_{\mathrm{i}\mathrm{n}}\times\:D}\end{array}$$

where *\:N=15* is the number of farmland units, $\:{T}_{\mathrm{i}\mathrm{n}}=14\:$ is the input sequence length, and *\:D=18* is the number of input features at each time step. The prediction target is represented as:

3 $$\:\begin{array}{*{20}c} {Y \in \: {\mathbb{R}} ^{{N \times \:T_{{{\mathrm{out}}}} }} } \\ \end{array}$$

where $\:{T}_{\mathrm{o}\mathrm{u}\mathrm{t}}=7$ is the forecast horizon.

#### Calculation of reference crop evapotranspiration (ET_0_) and crop evapotranspiration (ETc)

ET_0_ is calculated as follows^27^:

4 $$\:\begin{array}{*{20}c} {ET_{0} = \frac{{0.408\Delta \:\left( {R_{n} - G} \right) + \gamma \:\frac{{900}}{{T + 273}}u_{2} \left( {e_{s} - e_{a} } \right)}}{{\Delta \: + \gamma \:\left( {1 + 0.34u_{2} } \right)}}} \\ \end{array}$$

where *Δ* is the slope of the saturation vapor pressure–temperature curve (kPa/°C),*R*_*n*_ is net radiation (MJ/m²/day), *G* is soil heat flux (MJ/m²/day), approximated as 0 at the daily scale, *γ* is the psychrometric constant (0.067 kPa/°C), *T* is mean daily temperature (°C); *u*_*2*_ is wind speed at 2 m (m/s), *e*_*s*_ is the saturation vapor pressure (kPa), derived from maximum and minimum temperatures and *e*_*a*_ is actual vapor pressure (kPa), derived from relative humidity and *e*_*s*_.

Crop evapotranspiration is calculated as follows^28^:

5 $$\:\begin{array}{*{20}c} {ET_{c} = K_{c} \times \:ET_{0} } \\ \end{array}$$

where *ET*_*c*_ represents actual crop evapotranspiration, *ET*_*0*_ represents reference evapotranspiration, *K*_*c*_ is the crop coefficient, which varies with crop type and growth stage.

Crop coefficients (*Kc*) for maize and soybean at different phenological stages are listed in Table 4. Both *ET*_*0*_ and *ETc* are used in the framework as physically informed predictive variables and as reference indicators in the irrigation decision module.

**Table 4:** Crop coefficients for maize and soybean^29^.

Crop Type	Growth stage	Duration(days)	$K_c$	Water demand characteristics
Maize	Initial	20	0.30	Low water demand, dominated by soil evaporation
	Development	35	0.30→1.20 (linear)	Rapid increase in water use and leaf area expansion
	Mid-season	40	1.20	Peak demand, grain-filling stage
	Late-season	30	1.20→0.60 (linear)	Declining demand, crop maturity
Soybean	Initial	15	0.40	Low water demand
	Development	25	0.40→1.15 (linear)	Increasing water demand
	Mid-season	45	1.15	Peak demand, flowering, and pod setting
	Late-season	25	1.15→0.50 (linear)	Decreasing demand, crop ripening

#### Soil water balance

To link evapotranspiration with soil moisture dynamics, a simplified soil water balance equation is applied^30^:

6 $$\Delta \theta = P_{{eff}} + I - ET_{c} - D - R$$

where *Δθ* is the change in root-zone soil water storage (mm), *P*_*eff*_  is effective precipitation (mm), *I* is irrigation (mm), *ET*_*c*_ is crop transpiration (mm), *D* is deep percolation (mm) and *R* is surface runoff (mm). Effective precipitation was estimated using the USDA Soil Conservation Service (SCS) method: if monthly rainfall *P* ≤ 250 mm, *P*_*eff *_*= P × (125–0.2P) / 125*; if *P* > 250 mm, *P*_*eff *_*= 125.0.1P*.

Daily values were obtained by scaling monthly precipitation. Surface runoff (*R*) was estimated using the SCS Curve Number method, and deep percolation (*D*) occurred when root-zone soil moisture exceeded field capacity^31^.

These physically derived quantities (*ET₀*, *ETc*, and *Δθ*) play dual roles in the proposed framework. Firstly, they are incorporated as physically informed input features to the AI-STGNN encoder, providing prior knowledge on evapotranspiration dynamics and soil water balance processes. This enables the model to learn spatiotemporal patterns under physically meaningful constraints rather than relying solely on statistical correlations. Secondly, these variables are further utilized in the irrigation decision-making module (Sect.  2.4.9) as constraint indicators, ensuring that the predicted irrigation schedules satisfy physically admissible soil moisture ranges and crop water requirements. In particular, *ETc* serves as a reference for crop water demand, while *Δθ* constrains the allowable variation in root-zone soil water storage, thereby linking data-driven prediction with hydrological consistency.

### Research methods

#### Framework overview

**Fig. 1:**
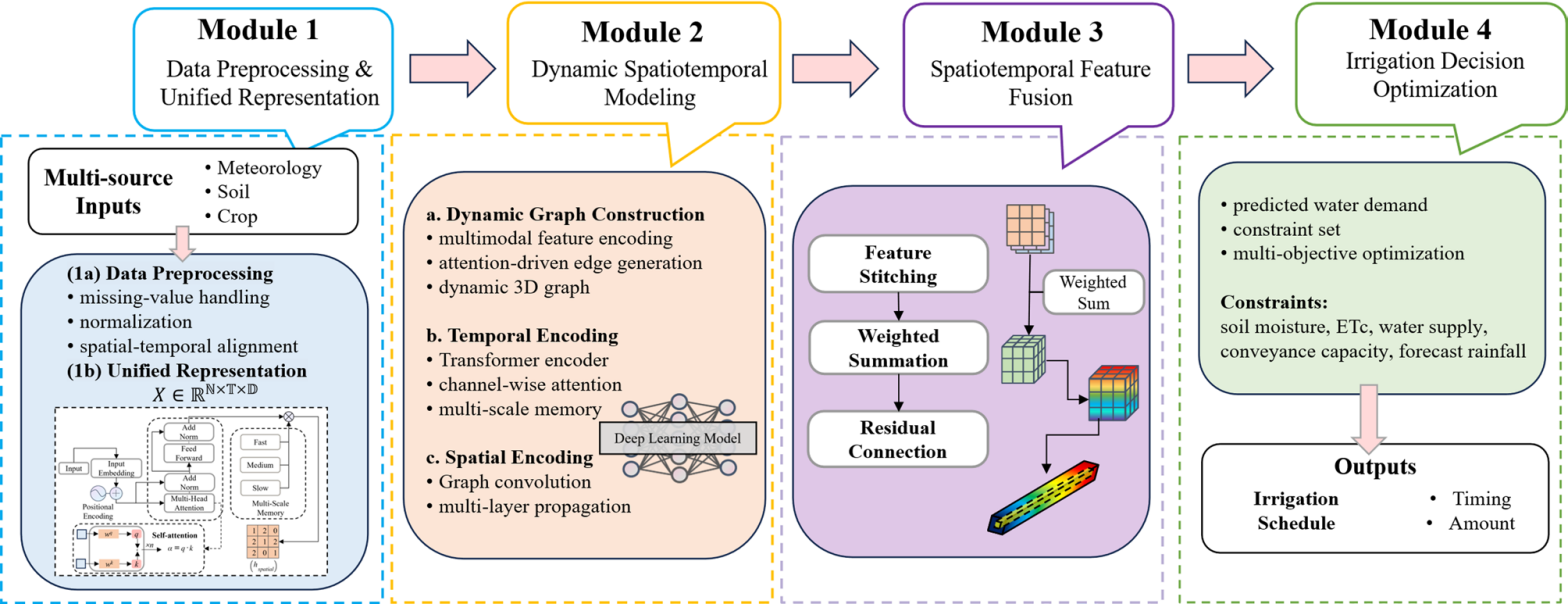
Modular architecture of the proposed AI-STGNN-based irrigation decision framework. The framework consists of four major modules: data preprocessing and unified representation, dynamic spatiotemporal modeling, spatiotemporal feature fusion, and irrigation decision optimization. Within the dynamic spatiotemporal modeling module, the framework further includes dynamic graph construction, temporal encoding, and spatial encoding.

As illustrated in Fig. 1, this study proposes an AI-STGNN-based irrigation decision framework for water-saving agriculture. The framework follows a unified pipeline consisting of four major modules. Firstly, meteorological, soil, and crop-related variables are integrated as multi-source inputs. After missing-value handling, normalization, and spatial–temporal alignment, these heterogeneous data are mapped into a unified feature representation for subsequent modeling. Secondly, the dynamic spatiotemporal modeling module combines dynamic graph construction, temporal encoding, and spatial encoding. Specifically, multimodal feature encoding and attention-driven edge generation are used to construct a dynamic 3D graph, while a Transformer-based temporal encoder with channel-wise attention and multi-scale memory captures temporal dependencies. In parallel, a graph convolutional network (GCN) extracts spatial dependencies among farmland units. Thirdly, the temporal and spatial representations are fused through feature alignment, weighted fusion, and residual connection to form an integrated spatiotemporal representation of farmland water processes. Finally, the irrigation decision optimization module converts the predicted water demand into operational irrigation schedules under multiple physical and management constraints, including soil moisture thresholds, evapotranspiration demand, water supply, conveyance capacity, and forecast rainfall. The resulting output provides irrigation timing and amount, forming a closed-loop pathway from multi-source sensing to decision support.

#### Data layer

The data layer provides unified acquisition, preprocessing, and representation of heterogeneous multi-source inputs for the proposed AI-STGNN-based irrigation decision framework. Three data modalities are integrated: meteorological variables, soil properties, and crop-related information, together capturing the main spatiotemporal dynamics of the farmland water cycle. Meteorological inputs include precipitation, temperature, relative humidity, wind speed, and solar radiation. Soil inputs include moisture content, electrical conductivity, texture, and organic matter from the surface to the root zone. Crop inputs include vegetation indices and field-observed growth variables. Detailed variable descriptions and data sources are provided in Table 2.

At the sample level, the processed inputs are mapped into a feature tensor:

7 $$X \in{\mathbb{R}}^{{T \times d}}$$

where *T* is the number of time steps (sequence length), and *d* is the feature dimension at each time step.

#### Dynamic layer

The dynamic graph learning module serves as the central mechanism for modeling time-varying farmland connectivity in the proposed framework. By updating graph topology and edge weights in response to evolving soil-moisture and meteorological conditions, it captures coupled interactions among soil, crop, and atmospheric processes. The module consists of two parts: a multimodal feature-encoding layer and an attention-driven edge-weight generation mechanism.

#### Multimodal feature-coding layer

Farmland observations are inherently multimodal. Soil electrical conductivity reflects subsurface water-related properties, NDVI captures crop growth status, and precipitation and evapotranspiration variables describe meteorological forcing. Directly concatenating such heterogeneous inputs can cause scale imbalance and modal conflict. Therefore, all variables were z-score standardized and passed through a Transformer encoder to learn a unified high-dimensional representation^32^. This encoder helps the model capture spatial patterns in soil properties, nonlinear relationships between vegetation dynamics and crop water demand, and temporal dependencies in meteorological sequences. The resulting representation provides the basis for constructing and updating the dynamic graph.

#### Attention-driven edge-weight generation

After the multimodal features are encoded, the edge-weight generation module computes dynamic relationships between farmland units. This module is designed to capture time-varying hydrological connectivity caused by soil moisture redistribution and meteorological disturbance. In irrigation systems, the strength of interaction between farmland units changes with local water conditions and weather forcing. Therefore, unlike static graph methods that use fixed adjacency relationships, the proposed framework updates edge weights dynamically according to soil moisture gradients and precipitation-related interactions. For each pair of connected nodes $\:i\:$and *\:j*, where the connection may represent either horizontal adjacency between neighboring farmland units or vertical linkage between soil layers, a hydrologically informed preliminary edge score is defined as:

8 $$\:\begin{array}{*{20}c} {e_{{ij,t}}^{0} = \alpha \:f_{{\theta \:}} \left( {\Delta \:\theta \:_{{ij,t}} } \right) + \beta \:g_{{\phi \:}} \left( {P_{{ij,t}} } \right)} \\ \end{array}$$

where $\:{e}_{ij,t}^{0}$ denotes the preliminary edge score between nodes $\:i\:$ and $\:j\:$ at time $\:t\:$; $\:{\Delta\:}{\theta\:}_{ij,t}=\mid\:{\theta\:}_{i,t}-{\theta\:}_{j,t}\mid\:\:$ is the absolute soil-moisture gradient between the two nodes; $\:{P}_{ij,t}\:$ denotes the precipitation-related forcing term shared by, or interpolated between, nodes $\:i\:$ and *\:j*; and $\:\alpha\:\:$ and $\:\beta\:$ are non-negative coefficients controlling the relative contributions of soil-moisture redistribution and meteorological forcing, respectively. The functions $\:{f}_{\theta\:}(\cdot\:)$ and $\:{g}_{\phi\:}(\cdot\:)$ are learnable nonlinear mappings parameterized by $\:\theta\:\:$ and $\:\phi\:$:

9 $$\:\begin{array}{*{20}c} {f_{{\theta \:}} \left( {\Delta \:\theta \:_{{ij,t}} } \right) = \sigma \:_{f} \left( {{\mathbf{W}}_{f} \Delta \:\theta \:_{{ij,t}} + {\mathbf{b}}_{f} } \right)} \\ \end{array}$$

10 $$\:\begin{array}{*{20}c} {g_{{\phi \:}} \left( {P_{{ij,t}} } \right) = \sigma \:_{g} \left( {{\mathbf{W}}_{g} P_{{ij,t}} + {\mathbf{b}}_{g} } \right)} \\ \end{array}$$

where $\:{\mathbf{W}}_{f}$, $\:{\mathbf{W}}_{g}$, $\:{\mathbf{b}}_{f}$, and $\:{\mathbf{b}}_{g}$ are learnable parameters, and $\:{\sigma\:}_{f}(\cdot\:)\:$and $\:{\sigma\:}_{g}(\cdot\:)\:$are nonlinear activation functions.

To incorporate feature-dependent node interactions, an attention coefficient is further computed as:

11 $$\:\begin{array}{*{20}c} {a_{{ij,t}} = \frac{{{\mathrm{exp}}\left( {{\mathrm{LeakyReLU}}\left( {{\mathbf{q}}^{T} [{\mathbf{z}}_{{i,t}} \left\| {\:{\mathbf{z}}_{{j,t}} } \right.]} \right)} \right)}}{{\sum {\:_{{k \in \:{\mathcal{N}}\left( i \right)}} } {\mathrm{exp}}\left( {{\mathrm{LeakyReLU}}\left( {{\mathbf{q}}^{T} [{\mathbf{z}}_{{i,t}} \left\| {{\mathbf{z}}_{{k,t}} } \right.]} \right)} \right)}}} \\ \end{array}$$

where $\:{\mathbf{z}}_{i,t}\:$ and $\:{\mathbf{z}}_{j,t}\:$ are the encoded feature representations of nodes *\:i* and *\:j* at time $\:t\:$, *∥* denotes vector concatenation, $\:\mathbf{q}$ is a learnable attention vector, and $\:\mathcal{N}\left(i\right)\:$ denotes the neighborhood of node $\:i\:$.

The final dynamic edge weight is then obtained by combining the hydrological edge score and the attention coefficient:

12 $$w_{{ij,t}} = a_{{ij,t}} \cdot e_{{ij,t}}^{0}$$

For numerical stability and comparability across nodes, the edge weights are normalized over the local neighborhood:

13 $$\:\begin{array}{*{20}c} {\mathop w\limits^{\sim } _{{ij,t}} = \frac{{w_{{ij,t}} }}{{\sum {\:_{{k \in \:{\mathcal{N}}\left( i \right)}} } w_{{ik,t}} + \varepsilon \:}}} \\ \end{array}$$

where $\:{\stackrel{\sim}{w}}_{ij,t}\:$ is the normalized dynamic edge weight used in the subsequent graph convolution, and $\:\epsilon\:$ is a small positive constant used to avoid division by zero.

Through this formulation, the proposed framework adaptively updates graph connectivity according to both soil moisture redistribution and precipitation-driven forcing. Intuitively, the gradient term strengthens edges when neighboring units are likely exchanging water, while the precipitation term strengthens edges when they are being driven by the same rainfall event. This allows the graph structure to better represent dynamic spatiotemporal interactions in farmland water systems and provides a more flexible basis for subsequent spatiotemporal modeling and irrigation decision optimization.

#### Temporal dynamics modeling layer

The temporal encoder employs a Transformer architecture enhanced with a channel-wise spatiotemporal attention mechanism and a multi-scale memory module to capture temporal dependencies in farmland water dynamics. The self-attention mechanism is defined as:

14 $$\:\begin{array}{c}\mathrm{A}\mathrm{t}\mathrm{t}\mathrm{e}\mathrm{n}\mathrm{t}\mathrm{i}\mathrm{o}\mathrm{n}\left(Q,K,V\right)=\mathrm{s}\mathrm{o}\mathrm{f}\mathrm{t}\mathrm{m}\mathrm{a}\mathrm{x}\left(\frac{Q{K}^{\mathrm{T}}}{\sqrt{{d}_{k}}}\right)V\end{array}$$

where Q, K, and V denote the Query, Key, and Value matrices, respectively, and $\:{d}_{k}$ denotes the dimensionality of the key vectors. The term $\:\sqrt{{d}_{k}}$ is introduced as a scaling factor to prevent excessively large dot-product values when the feature dimension increases, thereby improving numerical stability during attention computation.

To distinguish the heterogeneous impacts of meteorological drivers, temperature, precipitation, solar radiation, and wind speed are treated as separate channels within the attention mechanism. Temporal dependencies are modeled within each channel, while neighboring farmland units provide additional spatiotemporal context. The resulting inter-channel features are then adaptively weighted to identify the dominant meteorological drivers under different conditions. To capture both gradual evolution and abrupt disturbances, a multi-scale memory module is further incorporated into the temporal modeling architecture. The fast channel responds to short-term shocks such as heavy rainfall or heatwaves, the medium-scale channel captures daily-to-weekly fluctuations, and the slow channel models seasonal trends and long-term crop water use. These memory representations are integrated with the Transformer outputs to form a unified temporal representation. Through these designs, the temporal dynamics module captures long-term trends, short-term fluctuations, and heterogeneous meteorological influences, providing temporal support for irrigation water-demand prediction under varying environmental conditions.

#### Spatial dependency modeling layer

The graph convolutional spatial encoder was used to capture spatial dependencies among farmland regions and encode them as node-level features within a graph structure. Each soil unit—whether in the topsoil, root zone, or deep soil—was represented as a node, while edges denoted spatial or hydrological relationships between nodes. Through graph convolution, each node’s features were updated using information from its neighbors, allowing the model to effectively capture local spatial interactions.

A common graph convolution approach aggregates features from neighboring nodes to update the current node’s representation. Let $\:{\mathbf{h}}_{i}^{\left(l\right)}$ denote the feature representation of node *\:i* at graph convolution layer *\:l*. The graph convolution operation is defined as:

15 $${\mathbf{h}}_{i}^{{\left( {l{\text{ + }}1} \right)}} = \sigma \left( {\mathop \sum \limits_{{j \in{\mathcal{N}}\left( i \right)}} \tilde{w}_{{ij,t}} {\mathbf{W}}^{{\left( l \right)}} {\mathbf{h}}_{j}^{{\left( l \right)}} {\text{ + }}{\mathbf{b}}^{{\left( l \right)}} } \right)$$

where $\:\mathcal{N}\left(i\right)$ is the neighborhood of node *\:i*, $\:{\stackrel{\sim}{w}}_{ij,t\:}$ is the normalized dynamic edge weight between nodes *\:i* and *\:j* at time *\:t*, $\:{\mathbf{W}}^{\left(l\right)}\:$ and $\:{\mathbf{b}}^{\left(l\right)}\:$ are learnable parameters, and $\:\sigma\:(\cdot\:)$ is the nonlinear activation function. In this study, $\:\sigma\:(\cdot\:)$ is implemented as ReLU.

#### Spatiotemporal feature fusion mechanism

The spatiotemporal feature fusion mechanism combines the outputs of the graph convolutional spatial encoder and the Transformer temporal encoder to form a unified representation of farmland water processes. After feature extraction, the spatial and temporal representations are first mapped to the same latent space to ensure dimensional consistency.

Let $\:{h}_{\mathrm{spatial}}$ denote the output of the graph convolutional spatial encoder, and let $\:{h}_{\mathrm{temporal}}$ denote the output of the Transformer temporal encoder. The aligned features are concatenated as follows:

16 $$h_{{{\mathrm{combined}}}} = {\mathrm{concat}}\left( {h_{{{\mathrm{spatial}}}} ,h_{{{\mathrm{temporal}}}} } \right)$$

To balance the contributions of the two components, a learnable weighted fusion is then applied:

17 $$h_{{{\mathrm{fusion}}}} = {{\lambda }}h_{{{{spatial}}}} + \left( {1 - {{\lambda }}} \right)h_{{{\mathrm{temporal}}}}$$

where $\:{\uplambda\:}\in\:\left[\mathrm{0,1}\right]$ is a trainable parameter.

Through dimension alignment, concatenation, and weighted fusion, the model obtains an integrated spatiotemporal representation for subsequent irrigation demand prediction and decision-making.

#### Irrigation decision-making module

After modeling the spatiotemporal dynamics of the farmland system, the proposed framework further converts predicted crop water demand into actionable irrigation schedules. To achieve a balance between water conservation and crop productivity, an irrigation decision-making module is formulated as a multi-objective constrained optimization problem.

Let $\:{I}_{t}$ denote the irrigation amount applied at time step *\:t*, and let $\:\mathcal{H}=\{\mathrm{1,2},\dots\:,H\}\:$ denote the decision horizon, where $\:H=7\:$ days in this study. The irrigation decision module aims to reduce total irrigation input while maintaining adequate crop water supply. The optimization objective is defined as:

18 $$\mathop {{\mathrm{min}}}\limits_{{\left\{ {I_{t} } \right\}_{{t = 1}}^{H} }} J = \lambda _{1} \mathop \sum \limits_{{t = 1}}^{H} I_{t} - \lambda _{2} \mathop \sum \limits_{{t = 1}}^{H} B_{t}$$

where $\:J\:$ is the overall objective function, $\:{I}_{t}\:$ is the irrigation amount at time *\:t*, $\:{B}_{t}\:$ is the irrigation benefit indicator associated with crop water-demand satisfaction, and $\:{\lambda\:}_{1}\:$ and $\:{\lambda\:}_{2}\:$ are non-negative weighting coefficients used to balance water-saving and crop-production objectives.

The irrigation benefit indicator is calculated as:

19 $$B_{t} = min\left( {\frac{{\theta _{t}^{{{\mathrm{root}}}} - \theta _{{{\mathrm{min}}}} }}{{\theta _{{{\mathrm{opt}}}} - \theta _{{{\mathrm{min}}}} }}{\mathrm{,}}1} \right)$$

where $\:{\theta\:}_{t}^{\mathrm{r}\mathrm{o}\mathrm{o}\mathrm{t}\:}$ is the predicted root-zone soil moisture after irrigation, $\:{\theta\:}_{\mathrm{m}\mathrm{i}\mathrm{n}}\:$ is the lower soil-moisture threshold for avoiding water stress, and $\:{\theta\:}_{\mathrm{o}\mathrm{p}\mathrm{t}\:}\:$ is the target soil-moisture level for optimal crop growth. This formulation constrains $\:{B}_{t}\:$ to a maximum value of 1, indicating full satisfaction of crop water demand.

The optimization is subject to the following physical and operational constraints.

Firstly, the post-irrigation root-zone soil moisture must remain within the admissible range:

20 $$\theta _{{{\mathrm{min}}}} \le \theta _{t}^{{{\mathrm{root}}}} \le \theta _{{{\mathrm{max}}}} ,\forall t \in {\mathcal{H}}$$

where $\:{\theta\:}_{\mathrm{m}\mathrm{a}\mathrm{x}}\:$ denotes the upper admissible soil-moisture threshold, usually associated with field capacity or an upper management limit.

Secondly, irrigation and effective precipitation should satisfy the predicted crop water requirement:

21 $$I_{t} + P_{t}^{{{\mathrm{eff}}}} \ge ET_{{c,t}} ,\forall t \in {\mathcal{H}}$$

where $\:{P}_{t}^{\mathrm{e}\mathrm{f}\mathrm{f}\:}$ is effective precipitation and $\:E{T}_{c,t}\:$ is crop evapotranspiration at time *\:t*.

Thirdly, the root-zone soil water balance is constrained as

22 $$\theta _{{t + 1}}^{{{\mathrm{root}}}} = \theta _{t}^{{{\mathrm{root}}}} + P_{t}^{{{\mathrm{eff}}}} + I_{t} - ET_{{c,t}} - D_{t} - R_{t} ,\forall t \in {\mathcal{H}}$$

where $\:{D}_{t\:}$ and $\:{R}_{t}\:$ denote deep percolation and surface runoff, respectively.

Fourthly, irrigation must not exceed the available water supply:

23 $$0 \le I_{t} \le I_{{{\mathrm{max}},t}} ,\forall t \in {\mathcal{H}}$$

where $\:{I}_{\mathrm{m}\mathrm{a}\mathrm{x},t}\:$ is the maximum irrigation amount allowed by available water resources.

Fifthly, the irrigation amount must also satisfy the conveyance-capacity constraint:

24 $$I_{t} \le C_{t} ,\forall t \in{\mathcal{H}}$$

where $\:{C}_{t}$ denotes the maximum water delivery capacity of the canal or irrigation system at time t.

Finally, forecast rainfall is used to adjust irrigation decisions:

25 $$I_{t} \le \:I_{t}^{{{\mathrm{adj}}}} ,\:{\mathrm{if}}P_{{t:t + 3}}^{{{\mathrm{forecast}}}} \ge \:P_{{{\mathrm{thr}}}}$$

where $\:{P}_{t:t+3}^{\mathrm{f}\mathrm{o}\mathrm{r}\mathrm{e}\mathrm{c}\mathrm{a}\mathrm{s}\mathrm{t}\:}$ is the cumulative forecast precipitation over the next three days, $\:{P}_{\mathrm{t}\mathrm{h}\mathrm{r}}\:$ is the rainfall threshold for irrigation adjustment, and $\:{I}_{t}^{\mathrm{a}\mathrm{d}\mathrm{j}}\:$ is the adjusted upper bound for irrigation under expected rainfall.

Based on these constraints, candidate irrigation schedules are generated over the 7-day horizon. Infeasible schedules are discarded, and the feasible schedule with the lowest objective value is selected for implementation on the current decision day. The optimization is repeated daily as new AI-STGNN predictions and weather forecasts become available.

In the proposed framework, AI-STGNN provides predictions of soil moisture dynamics and crop water demand. Based on these predictions, the feasible irrigation space is first determined according to the above constraints. The optimization is implemented as a short-horizon rule-guided constrained search: for each decision day, candidate irrigation schedules over the 7-day forecast horizon are generated; schedules violating the soil-moisture, crop-water, soil-water-balance, water-supply, conveyance-capacity, or rainfall constraints are discarded; the objective function is evaluated on the remaining feasible candidates; and the candidate with the lowest objective value is selected for implementation on the current day. The search is then repeated after the forecast is updated, which preserves reproducibility while keeping the procedure computationally light enough for daily decision support. Through this process, the framework establishes a closed-loop linkage between spatiotemporal prediction and operational irrigation management.

### Experimental configuration and evaluation metrics

To evaluate model performance, the dataset was divided chronologically into training, validation, and test sets. The training set included data from 2015 to 2019 (five years, 1,826 days, 62.5% of total samples). The validation set used data from 2020 (one year, 366 days, 12.5%), and the test set covered 2021–2022 (two years, 730 days, 25%). This temporal partitioning ensured that the model had no access to test information during training and simulated real-world scenarios in which future conditions are inferred from historical records.

To further improve the robustness of evaluation under limited spatial coverage, the chronological split was complemented by temporal robustness checks during model development. In particular, model behavior was examined across sequential temporal segments to ensure that the reported performance was not overly dependent on a single partition of the time series. In addition, uncertainty in model performance was assessed by examining metric variability across temporally segmented subsets of the test period. The corresponding 95% confidence intervals provide an interval-based interpretation of predictive performance and complement the reported point estimates. For pairwise model comparison, statistical significance was evaluated using the Wilcoxon signed-rank test on matched absolute prediction errors. A significance level of *p* < 0.05 was adopted to determine whether the observed differences between AI-STGNN and the baseline models were statistically significant.

#### Model training configuration

For reproducibility, the complete architectural hyperparameters of the proposed AI-STGNN model are summarized in Table 5.

**Table 5:** Complete architectural hyperparameters of AI-STGNN.

Module	Sub-component	Configuration	Parameters
Multimodal Feature Encoder	Transformer	1 layer; d_model_ = 256; 4 heads; FFN dim = 1,024; dropout = 0.1	~ 723,000
	Positional encoding	Learnable; T_in_ = 14; d = 256	3,584
Temporal Encoder	Transformer	4 layers; d_model_ = 256; 8 heads; FFN dim = 1,024; dropout = 0.1	~ 2,885,000
	Fast memory channel	LSTM; hidden dim = 64; time window = 1–3 days	82,176
	Medium memory channel	LSTM; hidden dim = 128; time window = 3–7 days	197,120
	Slow memory channel	LSTM; hidden dim = 128; time window = 7–14 days	197,120
	Memory gate fusion	FC: [320 → 256]; ReLU	82,176
Spatial Encoder (GCN)	Layer 1	Input dim = 18 → hidden dim = 128; ReLU	2,432
	Layer 2	Hidden dim = 128 → 256; ReLU	33,024
	Layer 3	Hidden dim = 256 → 256; ReLU	65,792
Dynamic Edge Networks	f(·)	MLP: [256→128→64→1]; ReLU	41,537
	g(·)	MLP: [256→128→64→1]; ReLU	41,537
Channel-wise Attention	Per-channel FC	4 channels; FC: [256→64→1]; Sigmoid	66,052
Spatiotemporal Fusion	Dimension Alignment FC	[256→256] for spatial; [256→256] for temporal:	131,072
	Weighted sum	Scalar λ; learnable	1
	Residual projection	Identity	—
Prediction Head	FC	[512→256→128→T_out_ × N]; ReLU	164,975
	Total		4.7 M

The temporal encoder accounts for the largest proportion of model parameters, reflecting the importance of temporal pattern capture in agricultural water cycle forecasting.

Model training used the Adam optimizer with an initial learning rate of 0.001 and a weight decay coefficient of 1 × 10^− 5^. A ReduceLROnPlateau scheduler decreased the learning rate by 50% when the validation loss failed to improve for five consecutive epochs. The batch size was 64 and the maximum number of epochs was 200. Early stopping was triggered when the validation loss did not improve for 10 consecutive epochs. The loss function combined MSE and MAE with weights of 0.7 and 0.3, respectively. These weights were selected by grid search on the validation set. As shown in Table 6, the configuration (w_1_, w_2_) = (0.7, 0.3) achieved the lowest validation RMSE and the highest validation R² and was therefore used in all subsequent experiments. The relatively narrow performance range across candidate settings indicates that model performance is stable within this weighting interval. The higher weight on MSE was retained because large prediction errors are especially costly for irrigation scheduling during critical crop-growth stages, whereas the MAE term improves robustness to residual sensor noise.

**Table 6:** Validation set performance under different loss weight configurations.

w_1_ (MSE weight)	w_2_ (MAE weight)	Validation RMSE (vol%)	Validation MAE (vol%)	Validation *R*²
0.5	0.5	3.86	3.08	0.867
0.6	0.4	3.79	3.01	0.871
0.7	0.3	3.71	2.94	0.876
0.8	0.2	3.74	2.97	0.874
0.9	0.1	3.82	3.05	0.869

To address potential concerns regarding overfitting under limited spatial coverage, we clarify the training scale explicitly. The training period spans 1,826 days (2015–2019). With a sliding window of 14 input days and a 7-day forecast horizon (stride = 1), the chronological training set yields 1,806 graph sequences in total. Each sequence already contains all 15 farmland nodes, so these nodes should not be counted again as independent samples. Because each training sample is a graph tensor of size N × T × D = 15 × 14 × 18, the training set contains approximately 1.02 × 10^8^ scalar input values. This does not remove the need for caution about spatial representativeness or external validity, but it does show that the model is not being trained on only 15 isolated observations.

In addition, the graph convolutional layers share parameters across all spatial nodes, so each gradient update aggregates information from the full set of farmland units rather than learning node-specific weights. This parameter-sharing mechanism improves data efficiency and regularization by encouraging spatially transferable representations. Overfitting was further controlled through weight decay, early stopping, and adaptive learning-rate reduction based on validation loss. In the reported experiments, the training and validation losses converged without sustained divergence, which is consistent with stable optimization rather than severe overfitting.

#### Computational resources and evaluation metrics

Experiments were conducted on a high-performance server equipped with an NVIDIA RTX 3090 GPU with 24 GB VRAM, an Intel Xeon Gold 6248R CPU at 3.0 GHz with 48 cores, and 128 GB RAM. All models were implemented using PyTorch 1.12.0. A full training run required approximately 2.5 h. Model performance was evaluated using root mean square error (RMSE), mean absolute error (MAE), coefficient of determination $\:\left({R}^{2}\right)$, and Nash–Sutcliffe efficiency (NSE). The metrics are defined as follows:

26 $$RMSE = \sqrt {\frac{1}{n}\mathop \sum \limits_{{i = 1}}^{n} \left( {y_{i} \hat{y}_{i} } \right)^{2} }$$

27 $$MAE = \frac{1}{n}\mathop \sum \limits_{{i = 1}}^{n}{\mid }y_{i} - \hat{y}_{i} {\mid }$$

28 $$R^{2} = \left[ {\frac{{\mathop \sum \nolimits_{{i = 1}}^{n} \left( {y_{i} - \mathop y\limits^{} } \right)\left( {\hat{y}_{i} \mathop {\hat{y}}\limits^{} } \right)}}{{\sqrt {\mathop \sum \nolimits_{{i = 1}}^{n} \left( {y_{i} \mathop y\limits^{} } \right)^{2} } \sqrt {\mathop \sum \nolimits_{{i = 1}}^{n} \left( {\hat{y}_{i} \mathop {\hat{y}}\limits^{} } \right)^{2} } }}} \right]^{2}$$

29 $$NSE = 1 - \frac{{\mathop \sum \nolimits_{{i = 1}}^{n} \left( {y_{i} \hat{y}_{i} } \right)^{2} }}{{\mathop \sum \nolimits_{{i = 1}}^{n} \left( {y_{i} \mathop y\limits^{} } \right)^{2} }}$$

where $\:{y}_{i}\:$ is the observed value, $\:{\widehat{y}}_{i}$ is the predicted value, $\:\stackrel{\prime }{y\:}$ is the mean of the observed values, $\:\stackrel{\prime }{\widehat{y\:}}$ is the mean of the predicted values, and *\:n* is the number of observations. In this study, $\:{R}^{2}\:$ was calculated as the squared pearson correlation coefficient between observed and predicted values, whereas NSE was used as a hydrological efficiency metric that compares the residual variance of model predictions with the variance of the observations. NSE values closer to 1 indicate better agreement between predicted and observed values, whereas values below 0 indicate that the model performs worse than using the observed mean as a predictor.

## Results and discussion

### Comparative analysis

To comprehensively evaluate the performance of the proposed AI-STGNN framework, a suite of representative baseline models was selected to establish a rigorous comparison across different methodological paradigms. Specifically, the comparison included: (1) traditional machine learning methods, namely Random Forest (RF) and Support Vector Regression (SVR); (2) advanced deep learning models for sequential data, including Long Short-Term Memory (LSTM), Gated Recurrent Unit (GRU), and Temporal Convolutional Network (TCN); and (3) a state-of-the-art spatiotemporal graph model, namely Spatiotemporal Graph Convolutional Network (STGCN). The standard STGCN, which relies on a pre-defined static graph topology, served as a critical baseline to isolate the contribution of the proposed dynamic graph learning and 3D modelling mechanisms.

All models were trained and tested on the same dataset under identical conditions to ensure a fair comparison. Key hyperparameters for the deep learning baselines were standardized, including a two-layer LSTM/GRU with 128 hidden units, a four-block TCN, and a two-layer STGCN with Chebyshev graph filters k = 2. The static graph for STGCN was constructed based on spatial distance. This structured comparison was intended to quantitatively evaluate the relative advantages of AI-STGNN in prediction accuracy, robustness, and irrigation-oriented applicability.

To strengthen the methodological rigor of the comparison, statistical significance analysis was conducted on matched prediction errors between AI-STGNN and the competing baseline models. Pairwise Wilcoxon signed-rank tests showed that the performance improvements of AI-STGNN over the main baseline models were statistically significant at the 0.05 level. In addition, the reported error metrics were interpreted together with their confidence intervals, estimated from repeated temporal evaluations, to provide a more robust assessment of model performance variability across temporal subsets.

#### Overall performance comparison

As shown in Table 7, AI-STGNN produced the best overall performance across the reported metrics. Relative to RF and SVR, RMSE decreased by approximately 24.7% and 21.9%, respectively. Relative to LSTM and GRU, RMSE decreased by 12.5% and 10.8%, indicating that explicit spatial modeling improved predictive accuracy. Compared with STGCN, AI-STGNN reduced RMSE from 3.91 to 3.63 vol%, an absolute improvement of 0.28 vol% (7.2%). Pairwise Wilcoxon signed-rank tests on matched prediction errors indicated that the differences relative to the main baseline models were statistically significant at the 0.05 level, while the associated confidence intervals showed stable performance across temporal subsets.

In terms of MAE, AI-STGNN outperformed other models, reducing error by 14.1% and 8.6% relative to LSTM and STGCN. The coefficient of determination reached R² = 0.89, compared with 0.83 for the best baseline (STGCN), indicating a closer fit to the observed water-demand trends on the held-out test set. The NSE reached 0.88, outperforming all baselines and indicating better hydrological predictive agreement within the present evaluation setting.

**Table 7:** Performance comparison of different methods for soil moisture and water demand prediction.

Methods	RMSE (vol%)	MAE (vol%)	*R* ^2^	NSE	Training Time (h)	Parameters (M)
RF	4.82	3.67	0.72	0.69	0.3	-
SVR	5.14	3.91	0.68	0.64	0.5	-
LSTM	4.35	3.52	0.79	0.77	1.2	2.3
GRU	4.21	3.41	0.81	0.79	1.1	1.8
TCN	4.08	3.28	0.82	0.80	1.4	2.1
STGCN	3.91	3.15	0.83	0.82	1.8	3.2
AI-STGNN	3.63	2.88	0.89	0.88	2.5	4.7

RMSE and MAE are expressed as soil volumetric water content percentage points (vol%). Training time is measured on an NVIDIA RTX 3090 GPU. RF and SVR are ensemble methods without explicit parameter counts. Confidence intervals were estimated from temporally segmented test subsets, and pairwise Wilcoxon signed-rank tests were used to assess the statistical significance of model differences.

Table 7 demonstrated that graph-based methods generally outperformed pure temporal models, confirming the importance of spatial dependencies among farmland units. The dynamic edge-weight mechanism in AI-STGNN enabled the model to capture time-varying relationships influenced by soil moisture gradients and precipitation intensity, thereby maintaining high accuracy under complex weather conditions. Although AI-STGNN had a slightly higher training time (2.5 h) and parameter count (4.7 M), the performance gains justified the additional computational cost. Moreover, model inference required only ~ 0.5 s per sample, meeting real-time irrigation decision requirements.

The performance gains of AI-STGNN were primarily supported by its dynamic 3D graph structure, which learned to represent complex spatio-temporal correlations in the data, including patterns related to vertical soil moisture heterogeneity and inter-layer moisture movement. This was quantitatively supported by the ablation study (Table 11), where removing the 3D structure (AI-STGNN-2D) resulted in the largest performance drop (10.5% increase in RMSE), underscoring the importance of modelling the vertical dimension. Furthermore, the framework showed pronounced improvement (11.8% RMSE reduction) during crop developmental stages with rapidly changing water demand, which could be linked to the enhanced representation of root-zone moisture dynamics enabled by the 3D graph.

#### Performance under different meteorological conditions

To further evaluate model robustness, the test set was divided into three subsets based on precipitation intensity: dry, normal, and heavy rainfall periods. The classification criteria were: Dry period: 7-day cumulative rainfall < 5 mm and *ET*_*0*_ > 5 mm/day; Normal period: 7-day cumulative rainfall between 5 mm and 30 mm; Heavy rainfall period: 7-day cumulative rainfall > 30 mm or daily rainfall > 15 mm.

As presented in Table 8, AI-STGNN maintained stable performance across all conditions. During dry periods, when soil moisture changes are slow, all models achieved relatively high accuracy. AI-STGNN attained the lowest RMSE (2.95%), reflecting fine-grained modelling of slow variations. The channel-wise attention mechanism increased the weighting of temperature and radiation factors, accurately capturing evapotranspiration-driven soil drying.

Under heavy rainfall, the prediction errors of the baseline models increased sharply. LSTM’s RMSE rose from 3.21 vol% in dry periods to 5.47 vol% in heavy-rainfall periods, a 70% increase. In contrast, AI-STGNN’s RMSE increased from 2.95 to 4.28 vol% (45% increase), indicating better resilience to abrupt meteorological disturbances. This robustness is consistent with the combination of multi-scale temporal memory and dynamic graph learning, which allows the model to respond to sudden precipitation while preserving broader spatiotemporal context.

**Table 8:** Model performance under different meteorological conditions.

Methods	Dry RMSE (vol%)	Normal RMSE (vol%)	Heavy RMSE (vol%)	Relative Stability*
LSTM	3.21	4.18	5.47	0.70
GRU	3.12	4.02	5.29	0.70
TCN	3.05	3.89	4.95	0.62
STGCN	3.08	3.75	4.91	0.59
AI-STGNN	2.95	3.48	4.28	0.45

*Relative stability = (Heavy RMSE − Dry RMSE) / Dry RMSE; smaller values indicate more stable performance.

The results suggested that AI-STGNN remained comparatively stable under the tested meteorological variability. The channel-wise attention mechanism adaptively emphasized rainfall-related factors during heavy rainfall and temperature-radiation factors during dry periods, maintaining consistent accuracy. The stability index indicated that AI-STGNN’s performance fluctuation (45%) was lower than that of baselines (59–70%), supporting its use as an irrigation decision-support tool under diverse weather conditions. In addition, the multi-scale temporal memory component ensured stable performance during extreme events, limiting RMSE growth to about 45%, compared to 59–70% in other models. This illustrated AI-STGNN’s capacity to capture both short-term fluctuations and long-term hydrological trends within the present evaluation setting.

#### Performance across crop types and growth stages

To assess whether the proposed framework remains reliable under different physiological water-demand regimes, we further evaluated its performance across crop types and maize growth stages. This analysis is necessary because crop water demand is not stationary over the growing season. Different stages are associated with distinct rooting depths, evapotranspiration intensities, and sensitivities to soil moisture deficits, which may affect both prediction difficulty and irrigation relevance. In particular, the maize development and mid-season stages involve rapid changes in crop coefficient and water demand, making them more challenging for irrigation-oriented prediction. Therefore, stage-wise evaluation provides a more meaningful assessment of model applicability in irrigation practice than an aggregated test metric alone. It is also important to clarify the experimental setup underlying these analyses. The AI-STGNN model (and all baseline models) was trained as a single, unified model on a mixed dataset containing observations from both maize and soybean fields (training/validation period: 2015–2019), as described in Sect.  2.1 and 2.5. The overall performance results reported in Sect.  3.1.1 and 3.1.2 are based on evaluation using the entire crop-mixed test set (2021–2022), reflecting the model’s comprehensive predictive capability.

For the crop-specific analysis in this subsection, the trained model was applied to the same test set, and predictions were then grouped by the static crop-type variable (Table 2) to calculate separate performance metrics for maize and soybean, as shown in Table 9. To further examine stage-dependent behaviour, the maize test samples were partitioned into four growth stages—initial, development, mid-season, and late season—according to the crop coefficient (Kc) ranges listed in Table 4. The average prediction error for each stage was then computed, yielding the results shown in Table 10. This mixed-training, grouped-evaluation design preserves a unified learning setting while allowing a more detailed examination of the model’s adaptability to crop-specific and stage-specific water-demand dynamics.

**Table 9:** Prediction performance for different crop types.

Methods	Maize RMSE (vol%)	Maize *R*²	Soybean RMSE (vol%)	Soybean *R*²
LSTM	4.52	0.77	4.18	0.81
STGCN	4.08	0.82	3.74	0.84
AI-STGNN	3.78	0.88	3.48	0.90

All models achieved slightly higher accuracy for soybean than for maize, likely because soybean has a shallower root zone (0–40 cm) and simpler soil-water dynamics, whereas maize roots extend deeper (60–100 cm), increasing prediction difficulty. AI-STGNN’s 3D graph structure modeled vertical coupling among soil layers more effectively, reducing RMSE by 7.4% for maize and 7.0% for soybean relative to STGCN. Because irrigation scheduling is especially sensitive to prediction error during rapid shifts in crop water demand, the stage-level analysis below focuses on whether AI-STGNN retains this advantage during physiologically critical periods.

**Table 10:** Prediction performance across maize growth stages.

Stages	Duration (days)	K_c_ Range	AI-STGNN RMSE (vol%)	STGCN RMSE (vol%)	Improvement
Initial	20	0.30	3.12	3.45	9.6%
Development	35	0.30–1.20	4.25	4.82	11.8%
Mid-season	40	1.20	3.95	4.35	9.2%
Late	30	1.20–0.60	3.42	3.78	9.5%

The development stage exhibited the largest error (4.25 vol%) because rapid changes in crop coefficient (Kc = 0.30–1.20) caused pronounced shifts in water demand. Even in this most difficult stage, AI-STGNN outperformed STGCN by 11.8%. During the mid-season stage, when maize water demand peaks (8–10 mm/day), AI-STGNN maintained the lowest RMSE among the compared models (3.95 vol%), indicating that the model remained effective during the most irrigation-sensitive period.

### Ablation study

To quantify the contribution of key components, ablation experiments were conducted by removing or replacing specific modules in the AI-STGNN framework. Four core modules were examined: dynamic graph learning, 3D graph modelling, channel-wise meteorological attention, and the multi-scale memory module.

#### Ablation experiment results

Removing any core module led to a decline in model performance, confirming the effectiveness of each component (Table 11).

**Table 11:** Ablation experiment results.

Model variant	RMSE (vol%)	MAE (vol%)	*R* ^2^	NSE	RMSE Change
AI-STGNN	3.63	2.88	0.89	0.88	–
AI-STGNN-Static	3.89	3.12	0.85	0.84	↑7.2%
AI-STGNN-2D	4.01	3.24	0.83	0.82	↑10.5%
AI-STGNN-Unified	3.78	3.05	0.86	0.85	↑4.1%
AI-STGNN-Single	3.82	3.09	0.85	0.84	↑5.2%

#### Analysis of each module’s contribution

(1) *Dynamic Graph Learning (AI-STGNN vs. Static)*.

Replacing dynamic edge weights with static ones increased RMSE by 7.2%, highlighting the importance of modeling spatial dependencies arising from soil moisture gradients and precipitation intensity. Under heavy rainfall, the static variant reached an RMSE of 5.15 vol% compared with 4.28 vol% for AI-STGNN, corresponding to a 17% relative increase.

(2) *3D Graph Modelling (AI-STGNN vs. 2D)*.

Removing the 3D structure caused the largest degradation (RMSE + 10.5%). Different soil layers play distinct roles: the surface layer (0–10 cm) responds rapidly to rainfall, the root zone (10–40 cm) governs water uptake, and deeper soil (40–100 cm) provides storage and buffering. The 2D variant could not fully represent vertical water transfer, especially during drought. For deep-rooted maize, 3D modeling reduced RMSE by 10.6% relative to the 2D variant.

(3) *Channel-wise Meteorological Attention (AI-STGNN vs. Unified)*.

Removing channel separation increased RMSE by 4.1%, showing that different meteorological factors contribute unequally to evapotranspiration. Attention analysis (Fig. 3) indicated that temperature and radiation were dominant during droughts (67% of total weight), while precipitation was dominant during heavy rainfall (48%). Fixed weighting in the unified model reduced accuracy under extreme conditions.

(4) *Multi-Scale Memory Module (AI-STGNN vs. Single)*.

The single-scale variant increased RMSE by 5.2%, with the largest degradation under heavy rainfall (4.76 vs. 4.28 vol% RMSE; Table 12). The multi-scale design, which combines fast (1–3 days), medium (3–7 days), and slow (7–14 + days) channels, therefore appears necessary for capturing both short-term shocks and longer hydrological trends.

**Table 12:** Contribution of the multi-scale memory module under different conditions.

Condition	AI-STGNN RMSE (vol%)	Single RMSE (vol%)	Improvement
Dry	2.95	3.15	6.3%
Normal	3.48	3.72	6.5%
Heavy Rain	4.28	4.76	10.1%

#### Synergistic effects among modules

Dual-model ablation tests revealed that removing two modules simultaneously caused greater performance degradation (13.5–19.8%) than the sum of single-module effects, indicating strong synergy. Specifically, the dynamic-3D combination enabled real-time adjustment of vertical dependencies, dynamic-attention coupling aligned spatial structure with dominant weather factors, and 3D-memory integration enhanced spatiotemporal consistency. Overall, the ablation study demonstrated that dynamic graph learning, 3D modelling, channel-wise attention, and multi-scale memory jointly enabled AI-STGNN to capture complex spatiotemporal interactions in farmland water cycles and to improve water-demand prediction accuracy.

**Table 13:** Dual-module ablation results.

Variant	Removed Modules	RMSE (vol%)	Degradation
AI-STGNN	None	3.63	–
Variant-A	Dynamic + 3D	4.35	↑19.8%
Variant-B	Dynamic + Attention	4.12	↑13.5%
Variant-C	3D + Memory	4.28	↑17.9%

### Visualization analysis

#### Comparison of time series prediction performance

**Fig. 2:**
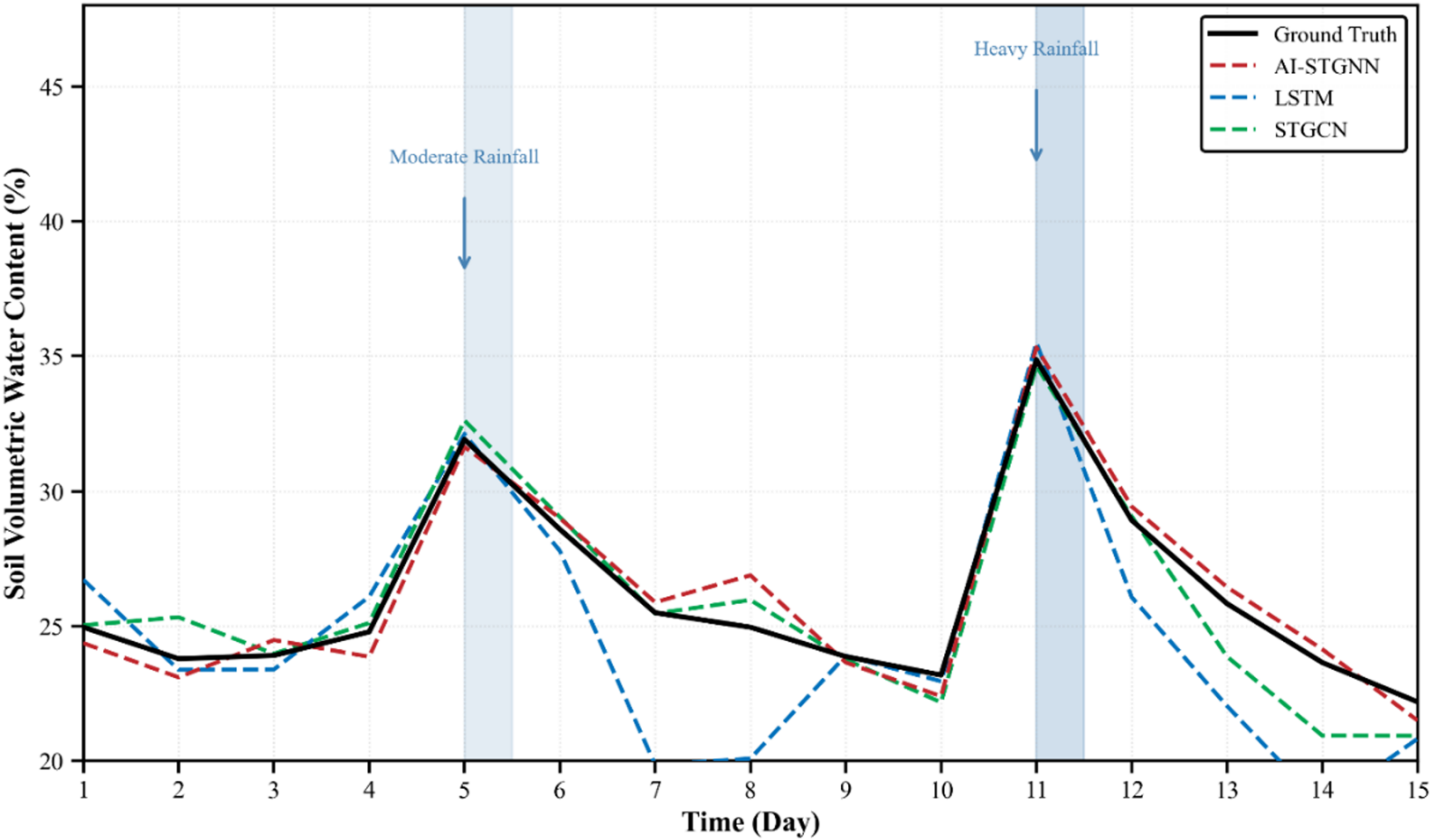
Comparison of soil moisture prediction results among different methods.

Figure 2 compares the predicted and observed root-zone soil volumetric water content for a representative farmland unit over a 15-day period in early July 2022, which included a moderate rainfall event on Day 5 and a heavy rainfall event on Day 11.

Before rainfall (Days 1–4), soil moisture remained near the crop water-stress threshold (24–25%). AI-STGNN closely matched the observations, with errors generally within ± 0.3%, while LSTM slightly underestimated and STGCN slightly overestimated the moisture level. During the two rainfall events, AI-STGNN more accurately captured the abrupt increases in soil moisture than the baseline models. After the moderate rainfall on Day 5 (18 mm), AI-STGNN predicted 30.8%, with an error of only 0.2%, whereas LSTM showed an evident lag and STGCN slightly underestimated the response. After the heavy rainfall on Day 11 (42 mm), AI-STGNN predicted 34.9% (0.1% error), compared with 32.5% for LSTM and 35.8% for STGCN. After rainfall, AI-STGNN also tracked the recession process more closely than the baseline models. From Days 6–10 and again from Days 12–15, it maintained smaller deviations during the gradual decline in soil moisture, whereas LSTM exhibited lagged responses and STGCN showed larger bias under dynamically changing moisture conditions.

Over the full 15-day period, AI-STGNN achieved an RMSE of 3.12 vol% and an MAE of 2.45 vol%, compared with 4.58 vol% and 3.62 vol% for LSTM, and 3.85 vol% and 3.01 vol% for STGCN. This corresponds to RMSE reductions of 31.9% and 19.0% relative to LSTM and STGCN, respectively.

#### Analysis of channel-wise meteorological attention weight evolution

**Fig. 3:**
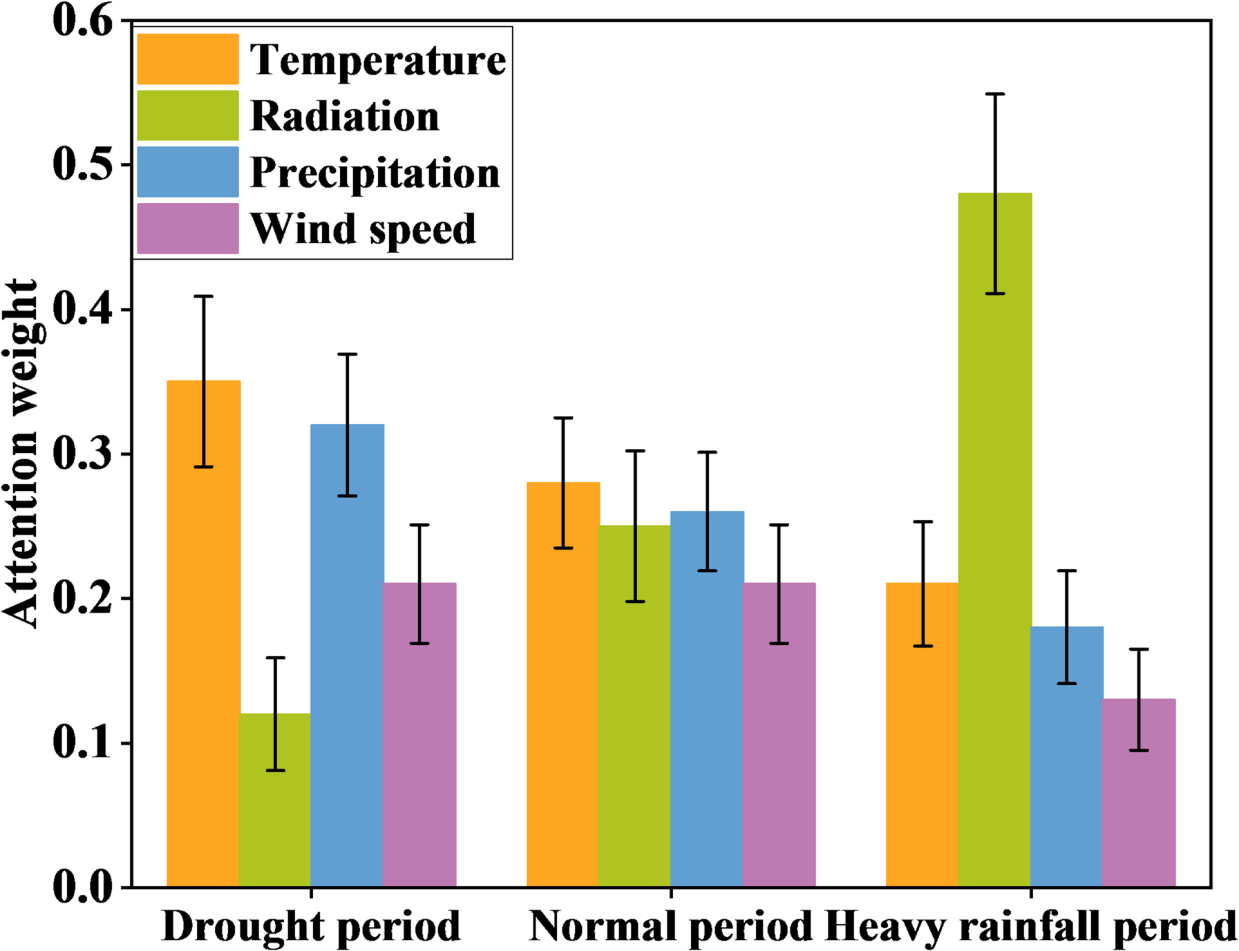
Channel-wise attention weight distribution under different meteorological conditions.

Figure 3 illustrates the dynamic evolution and uncertainty of meteorological attention weights under different conditions. The analysis used 500 time steps from the test set: 150 drought samples, 250 normal samples, and 100 heavy-rainfall samples, following the classification in Sect. 3.1.2. For each condition, mean attention weights and 95% confidence intervals were calculated.

From drought to heavy rainfall, the attention weight of temperature decreased continuously by 40%, reflecting its physical role in evapotranspiration. During drought (*P* < 5 mm/week), soil moisture loss mainly occurred through crop transpiration and soil evaporation. Higher temperatures increased the saturation vapor pressure deficit (*Δe*), intensifying evapotranspiration. Consequently, the model assigned a higher weight to temperature under drought. In contrast, the attention weight of precipitation increased sharply, rising by 300% from drought to heavy rainfall. Under heavy rainfall, precipitation became the primary source of soil moisture, accounting for nearly half of the total moisture. The wider confidence interval (± 0.069) highlighted the stochastic nature of rainfall—variability in intensity, duration, and spatial distribution affected model reliance on precipitation. Radiation showed a gradual decline, similar to the temperature trend. Radiation provided the energy for evapotranspiration, so its importance was higher during drought (contributing 67% of the total weight together with temperature) and lower under heavy rainfall due to cloud cover. Wind speed remained stable during both drought and normal periods, but slightly decreased during heavy rainfall. Its effect on evapotranspiration was minor compared to temperature and radiation, consistent with its narrow confidence interval (± 0.035).

Figure 3 showed a crossover of attention weights during the normal period, marking a transition from drought to heavy rainfall. Temperature (0.28 ± 0.045) and precipitation (0.25 ± 0.052) reached equilibrium, after which precipitation rapidly surpassed temperature as the dominant factor, reflecting the shift from an “evapotranspiration-dominated” to a “precipitation-recharge-dominated” water cycle.

Statistical analysis of confidence intervals confirmed significant differences among factors. During drought, temperature’s confidence interval did not overlap with precipitation, indicating a significantly higher weight (*p* < 0.01). During heavy rainfall, precipitation [0.411, 0.549] dominated, with no overlap with other factors (*p* < 0.01). These statistical results confirmed that the meteorological channel-wise attention mechanism accurately identified dominant drivers under varying conditions. The channel-wise meteorological attention module enhanced interpretability by dynamically adjusting the relative importance of temperature, radiation, and precipitation. During rainfall events, the attention weight for precipitation increased by almost 300%, whereas during dry periods, temperature and radiation became dominant. This shift reflected the transition from evapotranspiration-driven to rainfall-recharge-driven regimes, consistent with recent attention-based evapotranspiration studies showing that attention mechanisms can help identify the relative importance of meteorological drivers across different climatic conditions^33^.

#### Practical implications for irrigation management

Drought period: Temperature and radiation dominated (67%), leading to rapid soil moisture depletion. Close monitoring was required, and irrigation should be triggered when soil moisture fell below crop stress thresholds. Irrigation amounts should consider expected cumulative evapotranspiration over the next 3–5 days(∑ET_c_). Normal period: Factor weights were balanced. Irrigation could be delayed if rainfall was forecast and soil moisture was adequate; otherwise, moderate irrigation should be applied. Heavy rainfall: Precipitation dominated (48%). Irrigation should be adjusted based on forecasts: delayed or cancelled if rainfall met crop needs to prevent leaching and root hypoxia; supplemented if actual rainfall was insufficient.

This attention-based decision framework provides a data-informed basis for irrigation adjustment, with the potential to promote more efficient water use and stable crop yields within the studied setting.

#### Analysis of edge weight evolution in dynamic graph structures

**Fig. 4:**
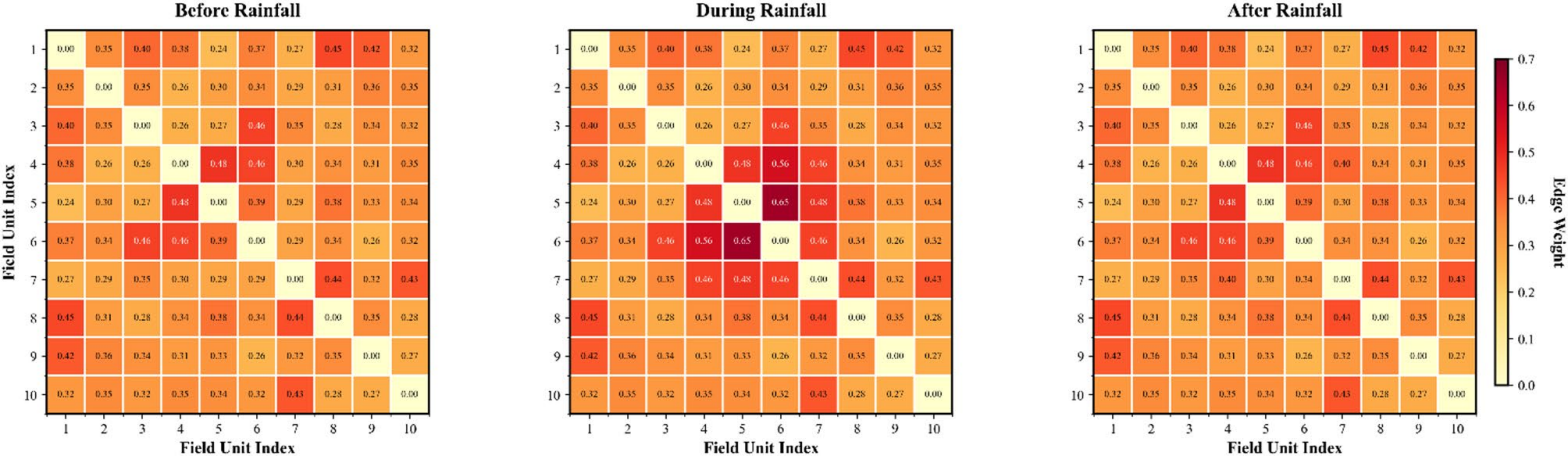
Heatmaps of dynamic edge-weight distributions across different time steps. The three panels show the normalized edge weights among field units at representative time steps corresponding to the three rainfall stages. In each panel, both axes denote field unit indices, and color intensity indicates the strength of inter-unit dependency, with darker colors representing larger edge weights.

Figure 4 presented heatmaps of dynamic edge-weight distributions at three representative time steps, shown as 10 × 10 matrices to illustrate the strength of dependencies among 10 farmland units (selected consecutively from 15 units for visualization simplicity). The time steps corresponded to Day 4 (pre-rainfall), Day 5 (during rainfall), and Day 6 (post-rainfall) in Fig. 2, capturing the edge-weight response to the rainfall event.

Pre-rainfall (Day 4): As shown in Fig. 4(a), before rainfall, edge weights among farmland units were relatively uniform, mostly ranging from 0.24 to 0.48 (light orange to orange in the heatmap). At this stage, edge weights were primarily determined by soil moisture gradients (the first term in the edge-weight formula). After four rain-free days, soil moisture in all units was low (24–25%), and inter-unit gradients were small (average ≈ 0.03), resulting in lower edge weights. Diagonal elements were 0.00 (white), indicating no self-connections. Spatial proximity slightly increased edge weights between adjacent units (e.g., units 1–2: 0.38; units 1–5: 0.26), reflecting the distance-decay effect.

During rainfall (Day 5): Fig. 4(b) shows that the overall edge-weight distribution remained close to the pre-rainfall state. The heatmaps at the three selected time steps retained similar large-scale spatial patterns, indicating that the dynamic graph preserved stable background connectivity while adjusting local weights in response to changing moisture and precipitation conditions. This behavior is consistent with a system in which adjacency is shaped by both persistent structural relationships and transient hydrological forcing.

Post-rainfall (Day 6): Fig. 4(c) showed that after rainfall, the edge-weight distribution remained coherent, with spatial patterns consistent with those observed before and during rainfall. Although soil moisture values changed over time, the model preserved stable learned spatial dependencies, highlighting the robustness of the dynamic graph learning mechanism.

These observations indicated that the dynamic graph learning mechanism was theoretically capable of adjusting edge weights according to soil moisture gradients and rainfall intensity. To enhance the spatial interpretability of the 10 × 10 edge-weight matrix in Fig. 4, we noted that the 10 field unit indices (1–10) corresponded to a continuously adjacent subset of our 15 study units, which were spatially arranged in a regular 50 m × 50 m grid pattern in the U.S. Midwest study area. Field units with adjacent indices (e.g., Unit 1 and 2, Unit 3 and 4) were physically contiguous and shared boundaries, meaning edge weights between indexed units in the matrix directly reflected interaction intensities between geographically adjacent field blocks.

Although the heatmaps remained broadly similar across the three selected time steps, this does not imply that the graph was static. Rather, it suggests that the underlying spatial connectivity of the study area was relatively stable over the selected event window, while the dynamic learning module adjusted edge magnitudes locally in response to rainfall and soil-moisture changes.

In addition, the model’s 3D graph structure incorporated vertical edge weights (not shown in Fig. 4) to represent water transfer between soil layers, enabling the capture of vertical moisture gradients and interlayer transmission processes, which were crucial for accurate prediction in deep-root crops.

#### Spatial distribution of prediction errors

**Fig. 5:**
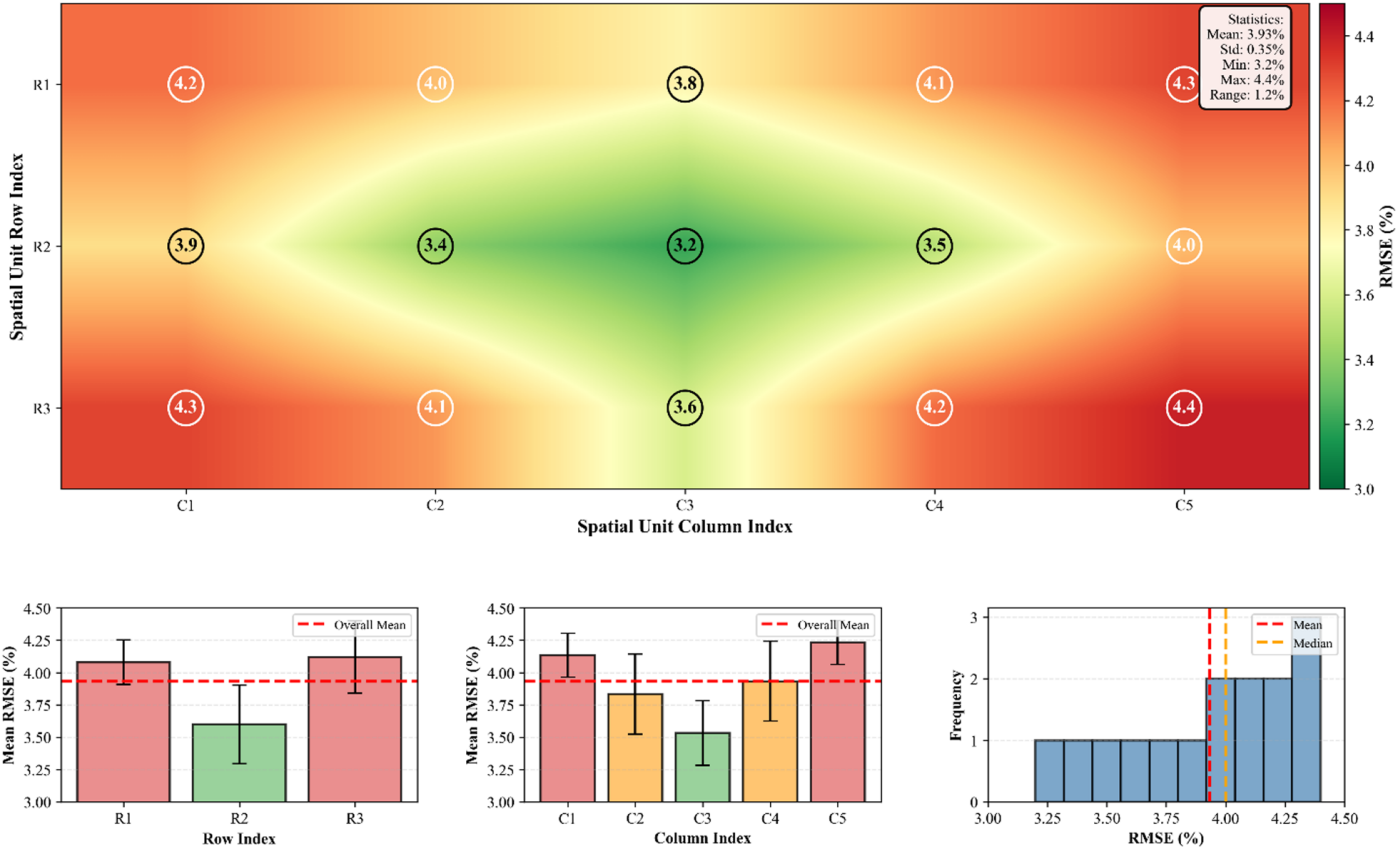
Spatial distribution of prediction errors for AI-STGNN across farmland units. The upper panel shows the RMSE distribution over the spatial grid, where color intensity indicates error magnitude. The lower panels summarize the mean RMSE by row, by column, and by overall frequency distribution, respectively. The dashed lines indicate the corresponding overall reference values.

To assess model accuracy across different locations, the spatial RMSE distribution across the 15 farmland units in the test set was visualized. Fig. 5 summarizes the corresponding heatmaps and descriptive statistics. The overall mean RMSE was 3.63 vol% (standard deviation 0.33 vol%), with individual errors ranging from approximately 3.0 to 4.4 vol%. Row 2 showed the lowest average error (~ 3.6 vol%), whereas rows 1 and 3 were slightly higher (~ 4.0–4.1 vol%). Column 3 had the lowest error (~ 3.5 vol%), whereas columns 1 and 5 were higher (~ 4.1–4.2 vol%).

These spatial variations likely arose from terrain effects, where slope differences influenced runoff and infiltration; boundary effects, because edge units had fewer spatial neighbors and stronger exposure to adjacent conditions; and soil heterogeneity, because differences in texture and organic matter affected water retention and transmission. This analysis highlights where further improvement is most needed. Future work could increase monitoring density near field boundaries or introduce explicit boundary-handling mechanisms, such as virtual nodes, to improve prediction accuracy in edge regions.

To compare the spatial error structure across models, we also summarized the test-set RMSE distributions of RF, LSTM, STGCN, and AI-STGNN in Table 14.

**Table 14:** Comparison of spatial RMSE (vol%) distributions of different models.

Methods	RF RMSE (vol%)	LSTM RMSE (vol%)	STGCN RMSE (vol%)	AI-STGNN RMSE (vol%)
Average (vol%)	4.82	4.35	3.91	3.63
Standard deviation (vol%)	0.51	0.47	0.41	0.33

Table 14 shows that AI-STGNN achieved the lowest mean spatial RMSE (3.63 vol%) and the smallest spatial standard deviation (0.33 vol%). This indicates that the edge-related error pattern observed in Fig. 5 is not unique to AI-STGNN; rather, it reflects a general difficulty at field boundaries and in topographically complex areas. Even under these conditions, AI-STGNN reduced both the magnitude and spatial variability of prediction error relative to the benchmark models.

### Practical application analysis

To evaluate practical utility, AI-STGNN-guided irrigation strategies were compared with conventional empirical irrigation methods. Three representative farmland areas were selected, covering different soil textures and crop types, with irrigation and yield recorded over two growing seasons (2021–2022).

#### Experimental site information

Table 15 summarized the characteristics of the three sites. Soil textures represented typical U.S. Midwest agricultural soils: sandy loam (Site A) with low water retention and frequent irrigation, loam (Site B) with balanced retention and infiltration, and clay loam (Site C) with high retention requiring infrequent irrigation.

**Table 15:** Basic Information of Experimental Sites.

Site	Coordinates	Area (ha)	Soil Texture	Sand/Silt/Clay (%)	Field Capacity (%)	Crop Type	Growing Season (days)
A	40.8°N, 96.5°W	12.5	Sandy loam	62/28/10	28	Maize	125
B	41.2°N, 96.8°W	10.8	Loam	42/38/20	33	Soybean	110
C	40.5°N, 97.1°W	15.2	Clay loam	35/40/25	36	Maize	125

#### Irrigation strategy design

Traditional Empirical Irrigation Method: (1) Cycle: Fixed irrigation every 7–10 days, disregarding real-time soil moisture and weather conditions. (2) Quota: Fixed amounts per event (e.g., Maize: 35 mm; Soybean: 30 mm), with minor adjustments based on general growth stage. (3) Basis: Historical farmer experience and predetermined growth stage calendars. (4) Implementation: Followed a fixed schedule (e.g., maize irrigated on days 10, 20, 30, 40… after sowing).

AI-STGNN-Guided Predictive Irrigation Strategy: (1) This strategy employed a multi-objective rule-based decision framework that utilized AI-STGNN forecasts to dynamically balance crop water needs against conservation goals. (2) Core Input – Water Demand Prediction: The system forecasted root-zone soil moisture and crop evapotranspiration (ET_c_) for the next 7 days ($hat :{\theta\:}(t+1:t+7)$ and $\:\widehat{E{T}_{c}}(t+1:t+7)$ ).

Decision Workflow: (1) Trigger: Irrigation was activated when the predicted soil moisture fell below the crop-specific stress threshold. (2) Optimization Framework: The timing and amount of irrigation were determined by solving a constrained decision problem aimed at minimizing water use while meeting crop demand. This process implicitly optimized the trade-off between water conservation and yield. (3) Decision Variables: Irrigation date and water volume. (4) Key Constraints: Soil moisture must remain within allowable bounds (lower stress threshold and upper field capacity); irrigation must satisfy predicted crop water deficit (ET_c_ - effective precipitation); available water supply and channel capacity; and weather forecasts (see below). (5) Weather Integration: Scheduled irrigation was deferred if significant rainfall (> 10 mm with > 70% forecast confidence) was predicted within the next 3 days and current soil moisture was adequate (> 10% above the stress threshold). (6) Implementation: Resulted in a dynamic, adaptive irrigation schedule updated daily based on the latest predictions and constraint satisfaction.

Both strategies were implemented using a drip irrigation system (assumed 90% efficiency). Each experimental site was divided into two equal subzones (50% each) to enable a direct, side-by-side comparison of the traditional and AI-STGNN-guided strategies.

#### Validation with field measurements

Eddy covariance (EC) and soil water-balance monitoring systems were used to estimate actual seasonal evapotranspiration. AI-STGNN maintained seasonal ET prediction errors within ± 3.1%, whereas the traditional empirical method underestimated seasonal ET by 6.9–7.3%. This difference is consistent with the fact that AI-STGNN responds to dynamic soil moisture stress, while the empirical method relies on fixed Kc values. The comparison between predicted and measured evapotranspiration is summarized in Table 16.

**Table 16:** Comparison of predicted and measured seasonal crop evapotranspiration.

Regions	Crops	Measured Cumulative (mm)	AI-STGNN Predicted Cumulative (mm)	Prediction Error (%)	Traditional Empirical Estimate Cumulative*	Empirical Error (%)
A	Maize	548	562	+ 2.6	510	-6.9
B	Soybean	425	438	+ 3.1	395	-7.1
C	Maize	572	585	+ 2.3	530	-7.3

*Traditional empirical estimates are based on fixed crop coefficients (Table 4) and the Penman–Monteith equation and do not account for dynamic soil water stress.

#### Comparison of irrigation strategy implementation

Table 17 compared the performance of AI-STGNN-guided irrigation with traditional empirical methods over two growing seasons. The AI-STGNN strategy consistently achieved water savings and yield increases across all three regions. Compared with conventional irrigation, AI-STGNN reduced water use by 12.3%, 15.7%, and 14.2% in regions A, B, and C, while increasing crop yields by 3.8%, 4.2%, and 3.5%, respectively.

**Table 17:** Water use and yield under different irrigation strategies (average of two growing seasons).

Regions	Strategys	Irrigation amount (mm)	Effective Rainfall (mm)	Total Supply (mm)	Yield (kg/ha)	WUE (kg/m³)	Water Saving (%)	Yield Increase (%)	Irrigation Events
A	Traditional	485	168	653	8520	1.31	-	-	14
A	AI-STGNN	425	168	593	8844	1.49	12.3	3.8	11
B	Traditional	512	195	707	9120	1.29	-	-	15
B	AI-STGNN	432	195	627	9503	1.52	15.7	4.2	10
C	Traditional	468	182	650	8340	1.28	-	-	13
C	AI-STGNN	402	182	584	8632	1.48	14.2	3.5	9

Effective rainfall calculated using USDA SCS method. Water Use Efficiency (WUE) = Yield / Total Supply, representing grain produced per unit water.

The water-saving effect of AI-STGNN can be explained by three mechanisms. Firstly, compared with the traditional fixed-schedule strategy, AI-STGNN triggered irrigation only when predicted soil moisture approached the crop-stress threshold, thereby avoiding unnecessary irrigation events. For example, in region A, the number of irrigation events decreased from 14 under the traditional strategy to 11 under AI-STGNN. Secondly, AI-STGNN incorporated 3–7-day rainfall forecasts into irrigation scheduling. Across two growing seasons, 12 irrigation events were delayed or cancelled, saving an estimated 360–480 mm of irrigation water. Thirdly, AI-STGNN determined irrigation amounts according to the estimated soil-water deficit rather than fixed quotas, which improved allocation efficiency and reduced cumulative water use.

The yield improvement under AI-STGNN can likewise be attributed to three mechanisms. First, the strategy maintained root-zone soil moisture above the crop-stress threshold during critical growth stages. For example, in region A, the average maize root-zone soil moisture during days 60–100 was 30.5% under AI-STGNN, compared with 28.2% under the traditional strategy. Second, AI-STGNN improved irrigation timing by anticipating short-term drought risk. In region A during 2022, a 12-day dry period was forecast, and AI-STGNN irrigated before soil moisture dropped below the stress threshold, whereas the traditional strategy responded later and produced mild water stress. Third, deficit-based irrigation amounts kept soil moisture within a more suitable range for root activity and crop growth, thereby supporting water uptake and nutrient utilization.

As a result, AI-STGNN increased water use efficiency (WUE) to 1.49, 1.52, and 1.48 kg/m³ in regions A, B, and C, respectively, compared with 1.31, 1.29, and 1.28 kg/m³ under the traditional strategy. This corresponded to improvements of 13.7%, 17.8%, and 15.6%, respectively, indicating that the proposed framework improved grain production per unit of irrigation water under the studied conditions.

Because only two growing seasons were available per region, we do not use this field comparison as the basis for strong formal significance claims. Instead, Table 18 reports the annual-yield summary as exploratory evidence, focusing on the direction and magnitude of the observed yield differences between the two irrigation strategies.

**Table 18:** Exploratory comparison of annual yield outcomes under the two irrigation strategies.

Regions	Strategys	Average yield (kg/ha)	Yield variability (SD)	Mean difference vs. traditional (%)	Evidence level	Interpretation
A	Traditional	8520	185	-	-	-
A	AI-STGNN	8844	162	3.8	Exploratory (2 seasons)	Higher mean yield; confirm with longer trials
B	Traditional	9120	210	-	-	-
B	AI-STGNN	9503	188	4.2	Exploratory (2 seasons)	Higher mean yield; confirm with longer trials
C	Traditional	8340	195	-	-	-
C	AI-STGNN	8632	170	3.5	Exploratory (2 seasons)	Higher mean yield; confirm with longer trials

As shown in Table 18, AI-STGNN achieved higher mean yield than the traditional strategy in all three regions, with average gains of 3.5–4.2%. These differences are encouraging and directionally consistent across the study sites, but they should be interpreted descriptively rather than as definitive statistical proof.

Because the field comparison is limited to two growing seasons, the robustness and practical applicability of these yield gains still require broader verification. The current evidence should therefore be interpreted as preliminary support for the practical potential of the proposed strategy rather than conclusive proof of broad field effectiveness. Future studies should include longer time series, more replicates, multi-site experiments under diverse climatic and irrigation conditions, and direct comparison with commercial precision irrigation systems.

#### Robustness analysis under extreme weather events

The robustness of the AI-STGNN framework was further evaluated under extreme weather conditions. Over two growing seasons, the experimental fields experienced four heavy rainfall events (daily precipitation > 25 mm) and three drought events (no rainfall for over 10 consecutive days). The comparison of irrigation strategies under extreme weather conditions was shown in Table 19.

**Table 19:** Comparison of irrigation strategies during extreme weather events.

Event Type	Date	Traditional Response	AI-STGNN Response	Outcome Comparison
Heavy rain (32 mm)	2021-06-15	Irrigated 35 mm on 2021-06-14; post-rain soil oversaturated, causing 15 mm runoff	Predicted rainfall, canceled 06–14 irrigation, utilized natural rainfall	Saved 35 mm and avoided runoff
Heavy rain (28 mm)	2021-07-22	Irrigated 40 mm one day before rainfall; soil saturated, causing nutrient leaching	Delayed irrigation to 3 days after rainfall, supplied 15 mm based on actual deficit	Net water saving 25 mm
Drought (12 days)	2022-05-10 to 05–21	Irrigated 35 mm on 05–15; soil still reached stress threshold on 05–20	Predicted prolonged drought; irrigated 25 mm on 05–12 and 05–19 to maintain soil moisture	Avoided stress, yield loss < 1% vs. 2.5% for traditional
Drought (15 days)	2022-08-01 to 08–15	Irrigated 35 mm on 08 − 05 and 08–12; crop late-season demand decreased, causing over-irrigation	Irrigated 30 mm on 08−06 and only 20 mm on 08–13 based on reduced Kc	Saved 20 mm and avoided overwatering

During heavy rainfall events, traditional irrigation systems did not integrate rainfall forecasts, leading to over-irrigation and water waste. For instance, prior to the 32 mm rainfall event on 2021-06-15, traditional irrigation applied 35 mm the day before. Post-rain soil moisture reached 42% (above field capacity at 36%), resulting in ~ 15 mm of runoff and nutrient loss. In contrast, AI-STGNN, using a 3-day advance forecast (78% confidence), cancelled the planned irrigation and fully utilized natural rainfall, saving 35 mm.

During drought events, traditional irrigation followed fixed schedules, but single applications failed to maintain soil moisture above stress thresholds. For example, during the 12-day drought from 2022-05-10 to 05–21, traditional irrigation applied 35 mm on 05–15, but soil moisture fell to 18% (threshold 18.2%) by 05–20, causing mild stress and ~ 2.5% yield loss.

AI-STGNN, leveraging the multi-scale memory module, captured slow soil-water depletion trends and predicted drought persistence early (05–12). Pre-emptive irrigation of 25 mm on 05–12 and 05–19 maintained soil moisture above the stress threshold (20–25%), avoiding water stress and limiting yield loss to < 1%. Although total AI-STGNN irrigation (50 mm) exceeded traditional irrigation (35 mm), it prevented yield loss and improved overall benefit.

These cases illustrated AI-STGNN’s robustness under extreme weather: (1) Predictive: Integration of 7-day forecasts allowed anticipation of rainfall or drought, enabling pre-emptive irrigation adjustment. (2) Dynamic: Daily updates adjusted irrigation timing and amount based on evolving weather and soil moisture conditions. (3) Timely: The predictions helped identify when irrigation should occur before soil moisture crossed critical stress thresholds in the studied cases.

In summary, the combined evidence from comparative experiments, ablation studies, visualization analyses, and the two-season field comparison supports AI-STGNN as a technically grounded framework for water-demand prediction and irrigation decision support within the studied setting. The largest performance contribution comes from the dynamic 3D graph, while channel-wise meteorological attention and multi-scale temporal memory provide additional gains under heterogeneous weather conditions. The field results are encouraging, but they remain preliminary because operational validation is limited to three sites and two growing seasons. The framework should therefore be viewed as a promising approach within this dataset and field context rather than as a fully generalized solution.

### Theoretical implications and research gaps

Despite rapid advancements in GNN-based hydrological prediction, several methodological limitations remain. Most existing models rely on static or two-dimensional (2D) graph representations, which are insufficient to capture vertical soil heterogeneity and dynamic hydrological connectivity. In addition, meteorological inputs are often aggregated into unified representations, neglecting the heterogeneous and nonlinear contributions of individual drivers such as temperature, precipitation, radiation, and wind. Recent hierarchical spatiotemporal graph studies for weather forecasting have shown that explicitly modeling interactions among multiple meteorological variables can improve the representation of complex atmospheric dependencies. These limitations may reduce model robustness under extreme or rapidly changing environmental conditions^34^.

The AI-STGNN framework addresses these challenges through three coordinated methodological enhancements. First, a dynamic three-dimensional (3D) graph structure is introduced to explicitly represent vertical soil-layer interactions and time-varying spatial dependencies, enabling more realistic modelling of water transport processes. Second, a channel-wise attention mechanism is employed to disentangle the contributions of different meteorological variables, allowing the model to adaptively emphasize dominant drivers under varying climatic conditions. Third, a multi-scale temporal memory module is designed to simultaneously capture gradual soil moisture evolution and abrupt disturbances caused by extreme weather events.

These components are further integrated into a unified irrigation decision framework, linking spatiotemporal prediction with operational decision-making. Compared with conventional spatiotemporal models, this design improves the representation of nonlinear, nonstationary, and multi-scale dynamics in farmland water systems, leading to more stable and interpretable predictions.

From a practical perspective, the two-season field comparison suggests that AI-STGNN can improve irrigation management within the studied context. Relative to the traditional empirical strategy, the AI-STGNN-guided strategy reduced water use by 12.3–15.7%, increased crop yield by 3.5–4.2%, and improved water use efficiency by up to 17.8% at the three evaluation sites. These results are directionally consistent with recent studies on AI-assisted irrigation^13^, but they should be interpreted as site-specific evidence rather than proof of broad deployment readiness. Future work should focus on integrating AI-STGNN with process-based crop models and extending its applicability through cross-regional validation, transfer learning, and domain adaptation.

Despite the relatively large number of temporal training windows, the proposed framework remains limited in spatial coverage. The current evaluation is confined to 15 farmland units in the U.S. Midwest, a relatively homogeneous agro-climatic region dominated by corn-soybean systems. Although this setting provides a representative testbed for large-scale irrigated agriculture, it does not fully capture the broader diversity of climatic conditions, soil types, and irrigation systems encountered in other regions. When transferred to semi-arid, monsoonal, or otherwise contrasting environments, the model may face challenges related to shifts in input feature distributions, variations in crop coefficients (Kc), and differences in irrigation practices, which could affect both the learned spatiotemporal relationships and the effectiveness of the irrigation decision module. Future work should therefore focus on cross-regional validation, multi-site experiments, and the integration of transfer learning and domain adaptation techniques to improve the robustness, scalability, and practical applicability of the proposed framework under more heterogeneous real-world conditions.

## Conclusions

In this study, an AI-STGNN-based framework was developed to couple farmland hydrology with irrigation decision-making. Across the held-out 2021–2022 test period, AI-STGNN consistently improved water-demand prediction relative to the benchmark models, with the clearest gain over STGCN being a reduction in RMSE from 3.91 to 3.63 vol%. The model also remained more stable under heavy-rainfall conditions, and the two-season field comparison suggested that it can reduce irrigation water use while improving water use efficiency and mean yield in the studied setting. These conclusions, however, should be interpreted within clear limits. The broader dataset covers only 15 farmland units in a relatively homogeneous Midwest corn–soybean region, and the operational comparison is limited to three sites over two growing seasons. Consequently, external validity and long-term practical robustness still require multi-site, multi-year, and cross-regional verification. In addition, although the framework is physically informed through evapotranspiration and soil-water-balance variables, its predictive core remains data-driven, so deeper integration with process-based crop-hydrology models would improve physical interpretability. Future work should prioritize broader field validation, uncertainty quantification, higher-resolution remote-sensing inputs, and systematic tests of cross-crop and cross-region transferability. Within these limits, AI-STGNN provides a promising basis for dynamic water-demand prediction and irrigation optimization in the studied Midwest corn-soybean production setting.

## Data Availability

The original contributions presented in this study are included in the article. Further inquiries can be directed to the corresponding authors.
